# RUNX1 is expressed in a subpopulation of dermal fibroblasts and is associated with disease severity of systemic sclerosis

**DOI:** 10.1016/j.ard.2025.10.033

**Published:** 2025-12-11

**Authors:** Rezvan Parvizi, Zhiyun Gong, Helen C. Jarnagin, Diana M. Toledo, Tamar R. Abel, Dillon Popovich, Madeline J. Morrisson, Tammara A Wood, Sasha Shenk, Mrinal K. Sarkar, Olesya Plazyo, Poulami Dey, Anthony Coon, Jake M. Erba, Lam C. Tsoi, Pei-Suen Tsou, Monique E. Hinchcliff, Dinesh Khanna, Jonathan A. Garlick, Johann E. Gudjonsson, Patricia A. Pioli, Michael L. Whitfield

**Affiliations:** 1 Department of Biomedical Data Science, Geisel School of Medicine at Dartmouth, Lebanon, NH, USA; 2 Department of Molecular and Systems Biology, Geisel School of Medicine at Dartmouth, Hanover, NH, USA; 3 Department of Basic and Clinical Translational Sciences, Tufts University School of Dental Medicine, Boston, MA, USA; 4 Department of Dermatology, University of Michigan, Ann Arbor, MI, USA; 5 Division of Rheumatology, Department of Internal Medicine, University of Michigan, Ann Arbor, MI, USA; 6 Section of Rheumatology, Allergy and Immunology, Department of Medicine, Yale School of Medicine, New Haven, CT, USA; 7 Department of Microbiology and Immunology, Geisel School of Medicine at Dartmouth, Lebanon, NH, USA

## Abstract

**Objectives:**

The activation of runt-related transcription factor 1 (RUNX1) in fibroblasts has been implicated in wound healing and fibrosis; however, the role of RUNX1 in the fibrotic progression of the autoimmune disease systemic sclerosis (SSc) remains known.

**Methods:**

Leveraging gene expression, genome-wide DNA methylation, and single-cell resolution data of SSc skin and fibroblast, we analysed the impact of *RUNX1* dysregulation in SSc dermal fibrosis. RUNX1 function was subsequently assessed using siRNA, pharmacologic inhibition, and CRISPR knockout in 2-dimensional and 3-dimensional fibroblast cultures.

**Results:**

Analysis of gene expression in multiple cohorts demonstrated an association between the severity of dermal fibrosis and the expression levels of *RUNX1* in the skin of patients with SSc. Epigenomic analyses of methylation identified hypomethylated 5-Cytosine-phosphate-Guanine-3 (CpG) sites proximal to the *RUNX1* gene, implicating their potential role in the increased expression of *RUNX1*. Analysis of single-cell RNA-seq data from skin biopsies of individuals with SSc revealed that *RUNX1* is higher in subpopulations of fibroblasts enriched in SSc, which are believed to contribute to fibrosis. *RUNX1* CRISPR knockout resulted in reduced alpha smooth muscle actin expression. Inhibition of *RUNX1* activity caused a reduction in fibroblast activation, contraction, extracellular matrix components, and proliferation rates, including a reduction in *SFRP4, LUM*, and *COL1A1*.

**Conclusions:**

This study is the first to demonstrate a potential role for *RUNX1* in the pathogenesis of SSc dermal fibrosis. RUNX1 is associated with more severe SSc fibrosis and is associated with a subpopulation of dermal fibroblasts implicated in fibrosis.

## INTRODUCTION

Dermal fibrosis is a major clinical manifestation of systemic sclerosis (SSc) [1,2], which is the result of uncontrolled deposition of extracellular matrix (ECM) by fibroblasts residing in the skin [3]. As dermal fibrosis progresses, patients with SSc experience skin thickening, distortion, and tightening that can limit joint movement and cause significant discomfort [4]. The collective crosstalk between dermal transcriptional patterns, signalling pathways, and epigenetic alterations contributes to fibrotic activation in SSc [3,5]. The modified Rodnan Skin Score (mRSS) is commonly used to quantify the degree and progression of dermal fibrosis and the severity of SSc. There are 2 clinical types of the disease, based on the extent of skin involvement: limited cutaneous SSc (lcSSc) and diffuse cutaneous SSc (dcSSc) [4]. We have developed a molecular classification system based on gene expression changes observed in lesional and nonlesional skin biopsies. This classification categorises patients with SSc into 4 subtypes: inflammatory, fibroproliferative, normal-like, and limited [6]. Through network analyses of transcription factor (TF) activity, we previously identified the *runt-related transcription factor 1* (*RUNX1*) as a key regulator in SSc but its function in SSc was not characterised [7].

RUNX1 is a member of a family of DNA-binding TFs that partners with a constitutively expressed core-binding factor subunit-*β* to form an active heterodimer that regulates the expression of downstream genes. The *RUNX1* gene is located on chromosome 21 and encodes 3 isoforms (a, b, and c) that differ in their N-terminal coding sequences [8]. Expression of *RUNX1* is tightly regulated by transcriptional, translational, and posttranslational mechanisms. Significantly, several studies have documented crosstalk between RUNX1 and profibrotic signalling pathways, including transforming growth factor-*β* (TGF-*β*) [9], Nuclear Factor kB (NF-*κ*B) [10], and Wnt signalling [11]. While the importance of RUNX1 in most haematopoietic cell lineages has been characterised in detail [12], its role in fibroblasts and dermal fibrosis more broadly remains poorly understood.

*RUNX1* is overexpressed in a range of human cancers that are characterised by fibrosis [13–16], including gliomas. There is a direct association between *RUNX1* levels, tumour grade, and the expression of ECM-related genes such as *Fibronectin 1* (*FN1*), *collagen type IV alpha 1 chain* (*COL4A1*), and *Lumican* (*LUM*), which are implicated in the pathogenesis of glioblastoma [17]. While RUNX1 is involved in wound healing, as demonstrated by a recent spatial multiomic analysis showing high RUNX1 activity in inner wound mechanofibrotic fibroblasts that differentiate into a strongly profibrotic cellular subset [18], it is also implicated in pathological fibrosis [19,20]. Additionally, *RUNX1* promotes the development of pulmonary fibrosis and pulmonary arterial hypertension [21,22] and mediates cardiac fibrosis following myocardial infarction [20,23,24]. Synovial biopsies from patients with rheumatoid arthritis and plaque biopsies from patients with atherosclerosis also identified *RUNX1* as a key upregulated TF [25], providing further support for RUNX1 in the pathogenesis of rheumatological and vascular disease.

In this study, we demonstrate for the first time *RUNX1* expression in a subpopulation of dermal SSc fibroblasts and investigate its role in regulating SSc disease severity. Using publicly available skin transcriptional datasets and analysing data from the skin of 200 patients with SSc, we characterise *RUNX1* expression patterns and epigenetic profile, then elucidate the effect of RUNX1 inhibition on ECM regulation and fibroblast function to establish a comprehensive understanding of its role in SSc dermal fibrosis.

## METHODS

A detailed description of the experimental methods, computational procedures, data processing, and statistical analyses can be found in the supplemental material.

## RESULTS

### RUNX1 expression is significantly higher in the skin of patients with diffuse SSc and is associated with a TGF-β fibroblast gene signature

**Figure 1.**
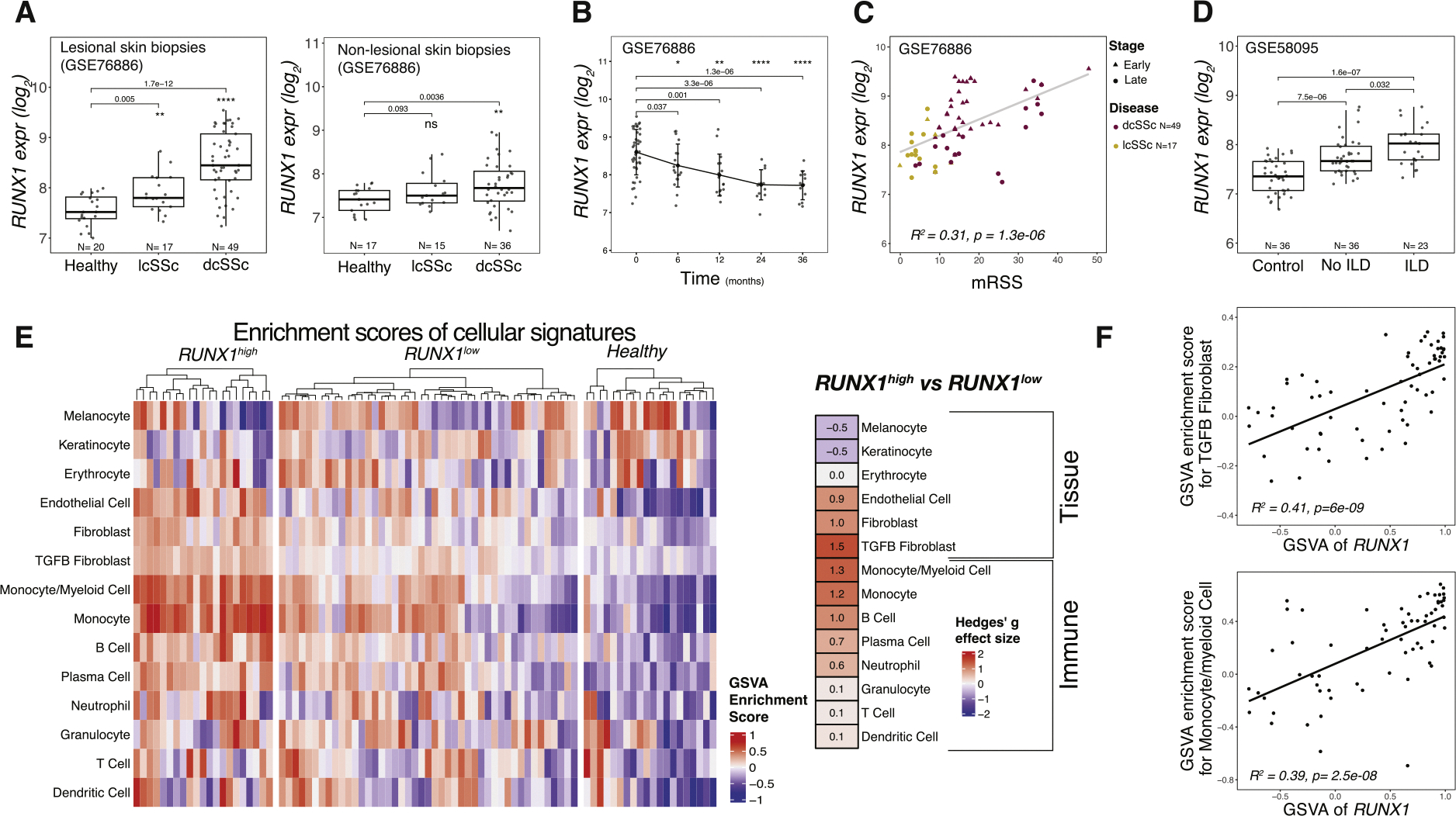
*RUNX1* expression is significantly higher in the skin of patients with dcSSc and is associated with TGF-*β* fibroblast and myeloid cell signatures. (A) *RUNX1* expression rate in forearm (lesional) skin biopsies of dcSSc (*N* = 49), lcSSc (*N* = 17), and healthy (*N* = 20) individuals and in flank (nonlesional) skin biopsies of dcSSc (*N* = 36), lcSSc (*N* = 15), and healthy (*N* = 17) individuals. (B) The expression rate of *RUNX1* over the course of 3 years (data presented for 0, 6, 12, 24, and 36 months). (C) Correlation between *RUNX1* expression and mRSS skin score at baseline for both lcSSc (yellow) and dcSSc (red) (*N* = 66); early-stage patients are shown as a triangle and late-stage patients as a circle. (D) *RUNX1* expression rate in healthy donors (*N* = 36), patients with ILD (*N* = 23), and patients without ILD (*N* = 36) at baseline. (E) GSVA enrichment scores of main cellular signatures in healthy (*N* = 20), patients with *RUNX1*^high^ (*N* = 21), and patients with *RUNX1*^low^ (*N* = 45). Hedge’s g effect size of *RUNX1*^high^ vs *RUNX1*^low^ is presented in the graph. (F) Correlation between the TGF-*β* fibroblast and monocyte and myeloid cell signatures with *RUNX1*. dcSSc, diffuse cutaneous SSc; GSVA, gene set variation analysis; ILD, interstitial lung disease; lcSSc, limited cutaneous SSc; mRSS, modified Rodnan Skin Score; RUNX1, runt-related transcription factor 1; TGF-*β*, transforming growth factor-*β*.

Our prior bioinformatic analyses predicted a role for the *RUNX1* TF in the inflammatory subset of SSc patients [7]. To validate and extend these findings, we analysed a larger cohort of skin biopsies from individuals with SSc and healthy controls to examine the distribution of *RUNX1* expression and to further investigate its correlation with disease severity [7]. Analysis of gene expression data showed significantly increased expression of *RUNX1* in lesional forearm skin biopsies from 86 individuals diagnosed with lcSSc and dcSSc [26]. *RUNX1* was also elevated in samples of nonlesional skin (samples collected from the flank of patients with SSc compared to healthy control nonlesional skin, Fig 1A), suggesting that the disease-specific activation of *RUNX1* is shared between early fibrotic skin and prelesional/prefibrotic skin biopsies. *RUNX1* expression was highest among individuals diagnosed with early dcSSc and decreased over the course of 3 years (Fig 1B, Supplementary Fig S1). Increased expression of *RUNX1* was subsequently confirmed in 5 additional publicly available datasets (GSE9285, GSE32413, GSE125362, GSE97248, and GSE58095), totalling more than 120 individuals with SSc [[6,27–31]] (Supplementary Fig S2A–E, Supplementary Table S1). At baseline, individuals with early disease (defined as biopsies taken less than 2 years since first onset of non-Raynaud’s symptoms) exhibited higher levels of *RUNX1* expression compared with those with a disease duration of greater than 2 years (Supplementary Fig S1). A significant positive correlation was found between *RUNX1* expression and mRSS in 2 separate cohorts [29,31] (*r*^*2*^ = 0.31, *P* = 1.3e-06; *r*^*2*^ = 0.25, *P* = 4.9e-05) (Fig 1C, Supplementary Fig S2F). *RUNX1* was highest among patients with SSc with a higher skin score at the site of biopsy (Supplementary Fig S2G) and those with interstitial lung disease (ILD) (Fig 1D) [7], indicating a potential association between disease severity and *RUNX1* expression levels.

We next set out to identify the cell-type-specific signalling pathways with which *RUNX1* expression was most closely associated. We performed gene set variation analysis (GSVA) to calculate the enrichment scores of fibroblasts, endothelial cells, keratinocytes, and immune cells [32] for 86 individuals at baseline (Supplementary Table S2). Patients with SSc with a high mRSS score (greater than 10) were divided into 2 groups: *RUN-X1*^high^, defined as *RUNX1* expression over 1 SD above the mean, and *RUNX1*^low^. The GSVA scores with the strongest enrichment in patients with *RUNX1*^high^ SSc were the TGF-*β*-activated fibroblast and monocyte/myeloid cell gene signatures (Fig 1E). Hedge’s g effect size was used to identify the magnitude of the difference between groups and confirmed strong enrichment of these pathways (effect sizes of 1.5 and 1.3, respectively). Similarly, evaluation of the Pearson’s correlation between the major cellular signatures and *RUNX1* demonstrated that TGF-*β*-activated fibroblast (*r*^*2*^ = 0.41, *P* = 6e-09) and monocytes/myeloid gene signatures (*r*^*2*^ = 0.39, *P* = 2.5e-08) had the highest correlations with *RUNX1* mRNA expression (Fig 1F).

### RUNX1 is expressed in a subpopulation of SSc-associated fibroblasts

**Figure 2.**
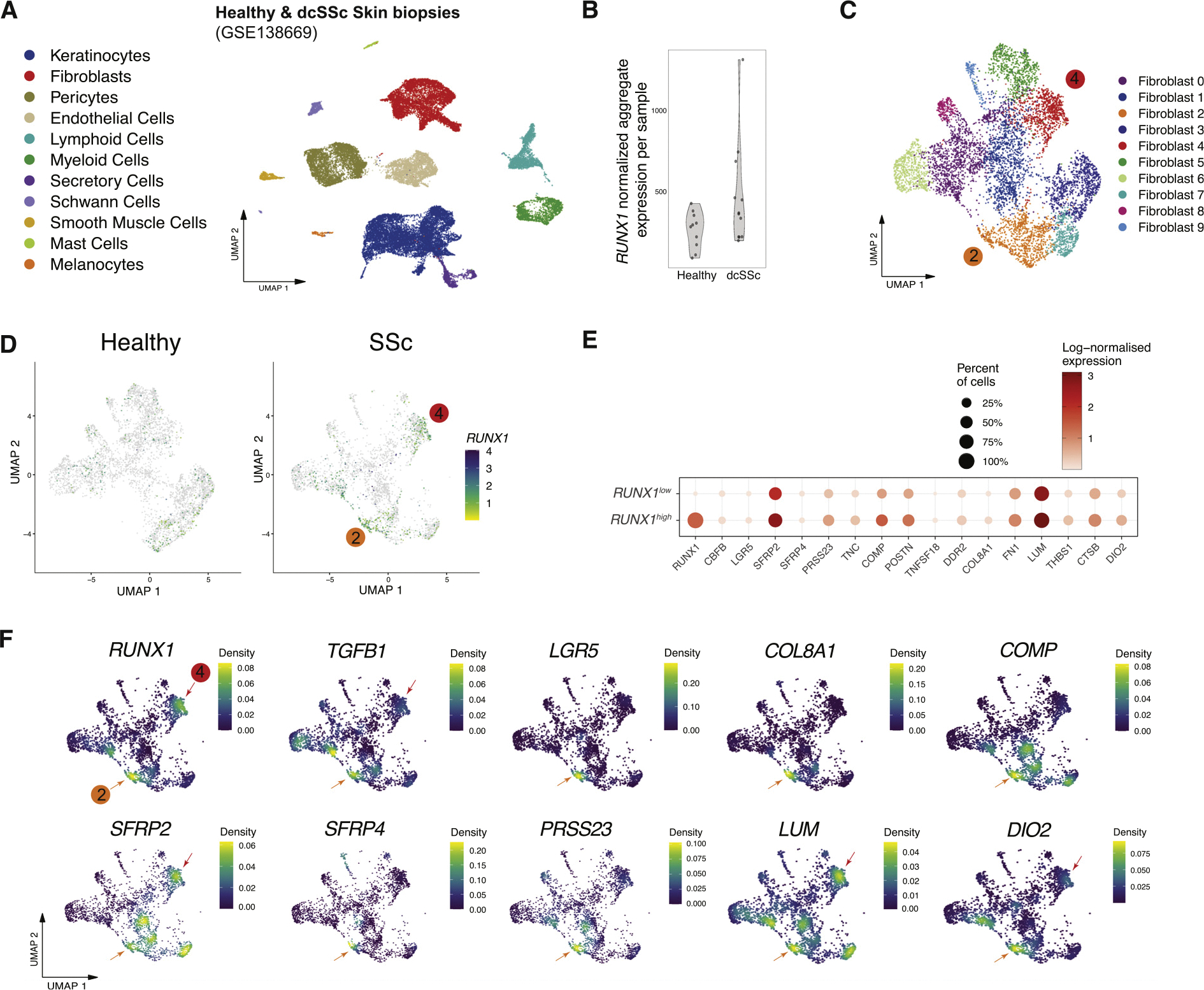
Enrichment of *RUNX1* in SSc-specific fibroblast subpopulations. (A) UMAP projection of cell types identified in the Tabib et al., [33] scRNA-seq of forearm skin biopsies. (B) *RUNX1*-normalised aggregate expression of 10 samples from healthy donors and 12 from patients with dcSSc. (C) UMAP projection of 10 fibroblast subpopulations (clusters 0–9). Two fibroblast populations of 2 and 4 are marked, which are enriched in SSc samples. (D) Feature plots of *RUNX1* expression in healthy and SSc fibroblasts. Two *RUNX1*-expressing fibroblast clusters are marked with their respective numbers. (E) The log-normalised expression rate of main differentially expressed genes between *RUNX1*^*high*^- and *RUNX1*^*low*^-expressing SSc fibroblasts. (F) Density plots of *RUNX1* and major SSc-relevant markers within SSc fibroblast subpopulations. Arrows indicate clusters 2 and 4 of SSc-specific subpopulations of fibroblasts. dcSSc, diffuse cutaneous SSc; RUNX1, runt-related transcription factor 1; scRNA-seq, single-cell RNA sequencing; SSc, systemic sclerosis; UMAP, uniform manifold approximation and projection.

To identify the fibroblast subpopulation(s) expressing *RUNX1*, we analysed publicly available single-cell RNA sequencing (scRNA-seq) data from 12 patients with dcSSc and 10 matched healthy controls (GSE138669) [33]. The skin biopsies in this study were collected from dorsal midforearm, and a single-cell suspension was created through enzymatic digestion. Figure 2A [33] shows the major cell types in a uniform manifold approximation and projection (UMAP) plot after data processing. The total *RUNX1* aggregate expression was then calculated and displayed in each sample. While heterogeneity exists between the patients, *RUNX1* was more highly expressed in dcSSc samples than in healthy control skin samples, in accordance with bulk gene expression analyses (Fig 2B). Notably, the patient with SSc exhibiting the highest *RUNX1* expression levels was in the early stage of the disease (duration of ~10 months; donor ID: SC189).

To examine *RUNX1* expression in the fibroblast subpopulations, the total fibroblast populations were clustered into 10 groups (clusters 0–9). As demonstrated in Figure 2C,D, *RUNX1* was enriched in SSc-enriched subpopulations 2 and 4. Comparison of *RUNX1*^*high*^-with *RUNX1*^*low*^-expressing single cells showed that the fibroblasts with high *RUNX1* expression levels also highly expressed fibrotic genes, including *FN1, LUM, POSTN, Cartilage Oligomeric Matrix Protein (COMP), COL8A1*, and *TNC* (Fig 2E). Analysis of markers identified in SSc-associated profibrotic fibroblasts from prior studies shows that *RUNX1* is expressed in *LGR5*^+^ and *TGF-β1*^+^ cells (Fig 2F). *LGR5*^+^ fibroblasts are associated with ECM degradation and skin remodelling/assembly genes [34]. Moreover, *RUNX1*-expressing fibroblasts are *SFRP2*^+^*/SFRP4*^+^/Col8A1^+^, indicating that these cells are myofibroblast progenitors [33]. Consistent with this result, these subpopulations of fibroblasts also express high levels of *COMP, PRSS23, LUM*, and *DIO2*.

### RUNX1 regulation is associated with the activation of TGF-β1 signalling in SSc fibroblasts

**Figure 3.**
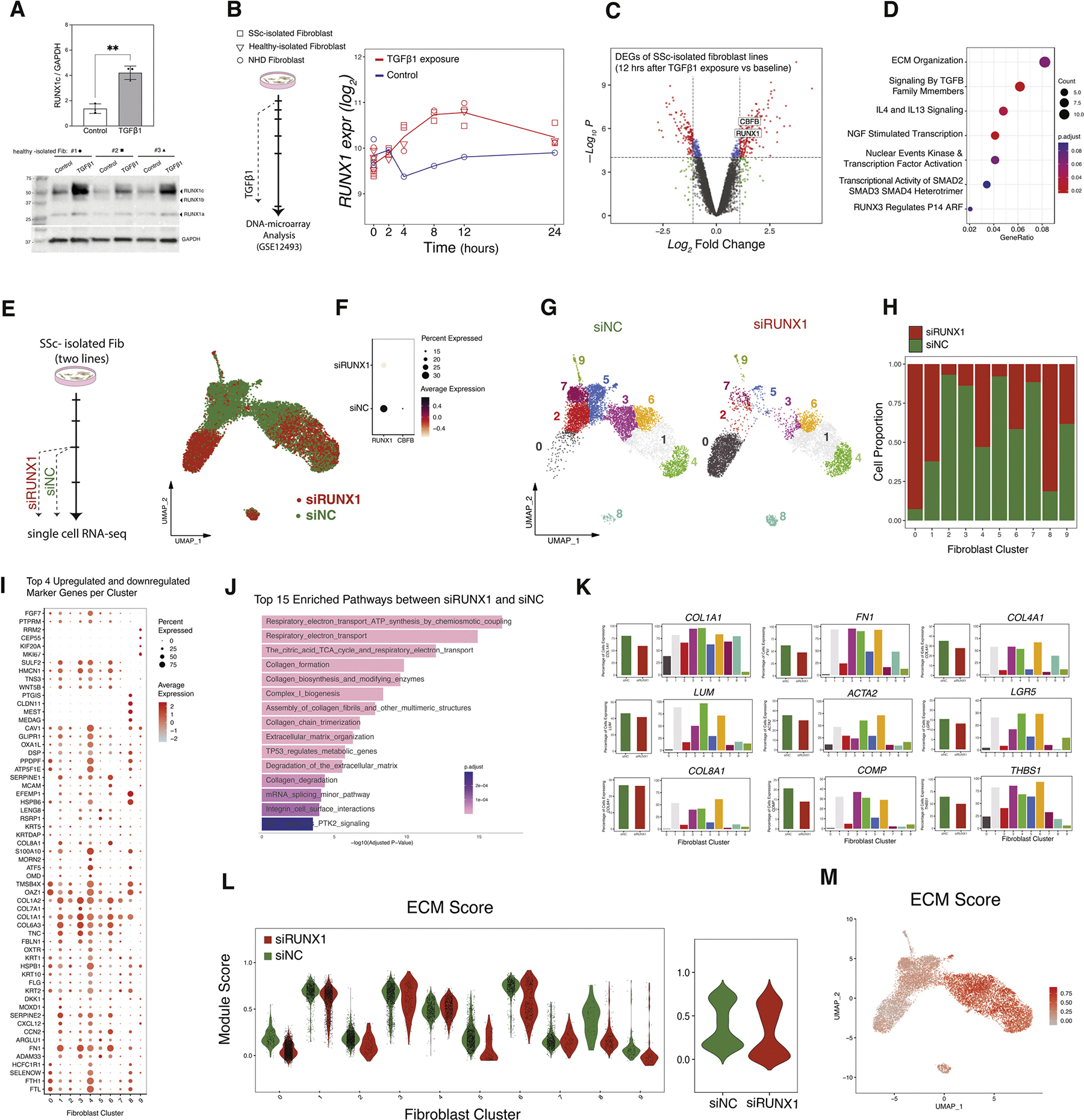
TGF-*β*1 increases the *RUNX1* expression in SSc fibroblasts and inhibition of *RUNX1* reduces ECM markers. (A) Western blot of 3 isolated fibroblasts lines treated with TGF-*β*1. The blot shows all isoforms of RUNX1a, b, and c that are overexpressed under the TGF-*β*1 stimulation. (B) Schematic graph illustrating the timeline for the culture and TGF-*β*1 treatment of dcSSc-isolated fibroblasts, matched healthy-isolated fibroblasts, and normal human dermal (NHD) fibroblast cells. *RUNX1* expression rate in samples treated with TGF-*β*1 (in red) vs control (in blue) for the 24 hours after exposure. (C) Volcano plot of differentially expressed analysis of the 2 SSc-isolated fibroblast lines at 12 hours after exposure vs the baseline. (D) The pathway analysis of Reactome gene sets shows the biological pathways and processes that are significantly represented within top DEG genes of SSc-isolated fibroblast lines 12 hours after TGF-*β*1 treatment vs the baseline. Data from B to D were obtained through publicly available data of GSE12493. (E) Schematic graph showing 2 lines of SSc-isolated fibroblasts treated with siRNA against RUNX1 (siRUNX1) and nontargeting control siRNA (siNC). (F) UMAP projection and dot plot of *RUNX1* and *CBFB* of the single-cell RNA-seq data. (G) UMAP of 10 fibroblast clusters (0–9) for siR-UNX1 and siNC. (H) Cell proportion of siRUNX1 and siNC per cluster. (I) Top 4 upregulated and downregulated marker genes per cluster. (J) Top 15 enriched pathways that are significantly represented across siRUNX1 and siNC (K) Bar plots showing the percentage of cells expressing *COL1A1, FN1, COL4A1, LUM, ACTA2, LGR5, COL8A1, COMP*, and *THBS1* per condition (red: siRUNX1, green: siNC) or per cluster. (L) Module score for extracellular matrix organisation pathway per cluster and per condition. (M) Feature plot of the ECM module score. dcSSc, diffuse cutaneous SSc; DEG, differentially expressed gene; ECM, extracellular matrix; RUNX1, runt-related transcription factor 1; siRUNX1, siRNA targeting RUNX1; SSc, systemic sclerosis; TGF-*β*, transforming growth factor-*β*; UMAP, uniform manifold approximation and projection.

To assess the regulation of *RUNX1* by the TGF-*β*1 signalling pathway, we investigated the expression of RUNX1 in fibroblasts after exposure to exogenous TGF-*β*1. TGF-*β*1 treatment of 3 isolated healthy fibroblasts significantly increased the expression of RUNX1 at the protein level (Fig 3A). We then analysed a previously generated DNA microarray dataset (National Center for Biotechnology Information Gene Expression Omnibus (NCBI GEO): GSE12493) consisting of 2 independent SSc fibroblast cell lines, 1 healthy control fibroblast cell line (isolated in parallel), and 1 normal human dermal (NHD) fibroblast cell line obtained from American Type Culture Collection (ATCC), treated with 50 pM TGF-*β*1 [35] (Fig 3A). As shown in Figure 3B, *RUNX1* expression was significantly induced by TGF-*β*1 (relative to vehicle alone treatment) as early as 4 hours after treatment, peaked at 12 hours, and remained elevated until the experimental endpoint at 24 hours. Differential gene expression analyses comparing SSc fibroblasts collected after 12 hours of TGF-*β*1 treatment with baseline showed significantly elevated levels of *RUNX1* and *CBFB* mRNA (Fig 3C). Analysis of the differentially expressed pathways using Reactome showed that the top pathways associated with high *RUNX1* expression in SSc fibroblasts after TGF-*β*1 exposure were ‘ECM organisation’, ‘signalling by TGF-*β* family members’, and ‘IL4 and IL13 signalling’ (Fig 3D).

To investigate whether inhibition of TGF-*β* would reduce *RUNX1* expression in SSc skin, we analysed data from a clinical trial of fresolimumab conducted by Rice et al [30]. Fresolimumab is a human monoclonal antibody that binds to and inhibits all 3 isoforms of TGF-*β* (TGF-*β*1–3). In this study, participants were divided into 2 treatment arms: 1 that received 2 low-dose infusions of fresolimumab and another that received 1 high-dose infusion. Midforearm skin biopsies were collected at baseline and 3, 7, and 24 weeks after treatment. While no significant changes were observed in samples analysed at 24 weeks post-treatment, expression of *Thrombospondin 1 (THBS1)*, which was used as a biomarker of TGF-*β* pathway activation, was decreased 3 and 7 weeks after treatment in patients who received high-dose fresolimumab. *RUNX1* expression decreased with fresolimumab treatment, paralleling *THBS1* expression (Supplementary Fig S3A). Analysis of the TGF-*β* fibroblast gene signature at baseline and 3 weeks after a single high-dose treatment with fresolimumab showed that TGF-*β*-associated genes had decreased expression (Supplementary Fig S3B). Consistent with inhibition of *RUNX1* and *THBS1*, expression of *CBFB, CCL2, COMP, TGFB1, ACTA2, COL4A1*, and *FN1* was attenuated in 5 of 7 patients that received high-dose fresolimumab (Supplementary Fig S3C). Collectively, these data imply that TGF-*β* signalling regulates the expression of *RUNX1* in SSc skin.

### Inhibition of RUNX1 reduces the ECM signature

To investigate the mechanism by which RUNX1 activity regulates SSc fibroblast activation, we isolated single-cell suspensions from SSc fibroblasts treated with siRNA targeting RUNX1 (siRUNX1) and a nontargeting control (siNC) as input for scRNA-seq (Fig 3E, Supplementary Table S3). Quality-controlled single-cell data yielded 7544 siRUNX1 and 5738 siNC cells, with an average of 1387 genes and 2768 transcripts per cell. Consistent with single-cell transcriptomic analyses of skin biopsies, we selected highly variable genes and performed UMAP-based dimensionality reduction and clustering (Fig 3E, Supplementary Fig S4A). As indicated in Figure 3F, *RUNX1* and *CBFB* expression were markedly reduced following knockdown (KD) in siRUNX1-transfected cells. Subsequent clustering analysis identified 10 distinct fibroblast subpopulations (Fig 3G,H). In contrast to fibroblast subpopulations observed in skin tissue, these clusters exhibited reduced functional heterogeneity, as reflected by the top differentially expressed markers in each cluster (Fig 3I). Notably, clusters 0 and 8 were predominantly composed of siRUNX1-derived cells (Fig 3H).

Cell cycle analysis revealed a decrease in the G2/M phase genes in siRUNX1 cells compared to siNC transfectants (Supplementary Fig S4B). Moreover, the small proliferative cluster 9 was primarily composed of siNC fibroblasts Supplementary Fig S5 and is identified with cell cycle markers such as *KIF20A, CEP55*, and *MKI67* (Fig 3I). This suggests that RUNX1 is essential for fibroblast proliferation *in vitro.* Differential expression analysis between siRUNX1 and siNC fibroblasts identified a substantial number of differentially expressed genes in the RUNX1 KD samples. Functional pathway analysis of the top 15 upregulated and downregulated pathways demonstrated significant alterations in collagen synthesis, degradation, assembly, and ECM organisation (Fig 3J, Supplementary Table S4). Accordingly, key molecules involved in ECM and fibroblast activation, including *ACTA2* (alpha smooth muscle actin [*α-*SMA]), *COL1A1, COL4A1, FN1, LUM, COL8A1, COMP*, and *LGR5* were all expressed at a lower percentage in siRUNX1 fibroblasts, particularly in clusters 0 and 8 compared with clusters 3, 4, and 6 (Fig 3K), suggesting RUNX1 promotes myofibroblast transition.

To illustrate the capacity for ECM involvement by different fibroblast clusters and the effect of RUNX1 KD, we compared the module score based on the gene set from the ECM organisation pathway in the Reactome database as well as other significantly altered pathways such as collagen formation, Tricarboxylic Acid Cycle (TCA) cycle, fibroblast activation and migration (Fig 3L,M, Supplementary Fig S4C,D). Fibroblasts transfected with siRUNX1 exhibited a decreased ECM module score compared with siNC fibroblasts, with the most pronounced reductions observed in clusters 0 and 8 (Fig 3L,M). This suggests that reduced RUNX1 activity via siRNA downregulates collagen gene expression, disrupts ECM organisation, and reduces the activated fibroblast subpopulations.

### Epigenetic dysregulation of RUNX1 in SSc fibroblasts

To evaluate the epigenetic state of *RUNX1* in SSc fibroblasts, we conducted genome-wide DNA methylation profiling using Illumina’s Infinium Methylation EPIC array to characterise fibroblasts in either 2-dimensional (2D) culture or in a 3-dimensional (3D) self-assembled (SA) skin-like tissue model culture [36,37]. Clinical characteristics of the patients with dcSSc and healthy donors are provided in Supplementary Tables S3 and S5. Two skin biopsies were collected from each donor, with 1 biopsy taken from forearm skin (lesional) and the other from flank skin (nonlesional). DNA was extracted from fibroblasts in 2D culture or in 3D SA tissues. The methylation EPIC array was used to assess cytosine methylation at over 850,000 CpG sites across the genome. Once processed, data were filtered based on the most variated methylated sites; 592 CpG sites passed this filter and were hierarchically clustered by beta values (Fig 3A, Supplementary Table S6). The beta value shows the degree of DNA methylation at a specific genomic locus, where 0 represents completely unmethylated CpG sites and 1 represents completely methylated CpG sites.

**Figure 4.**
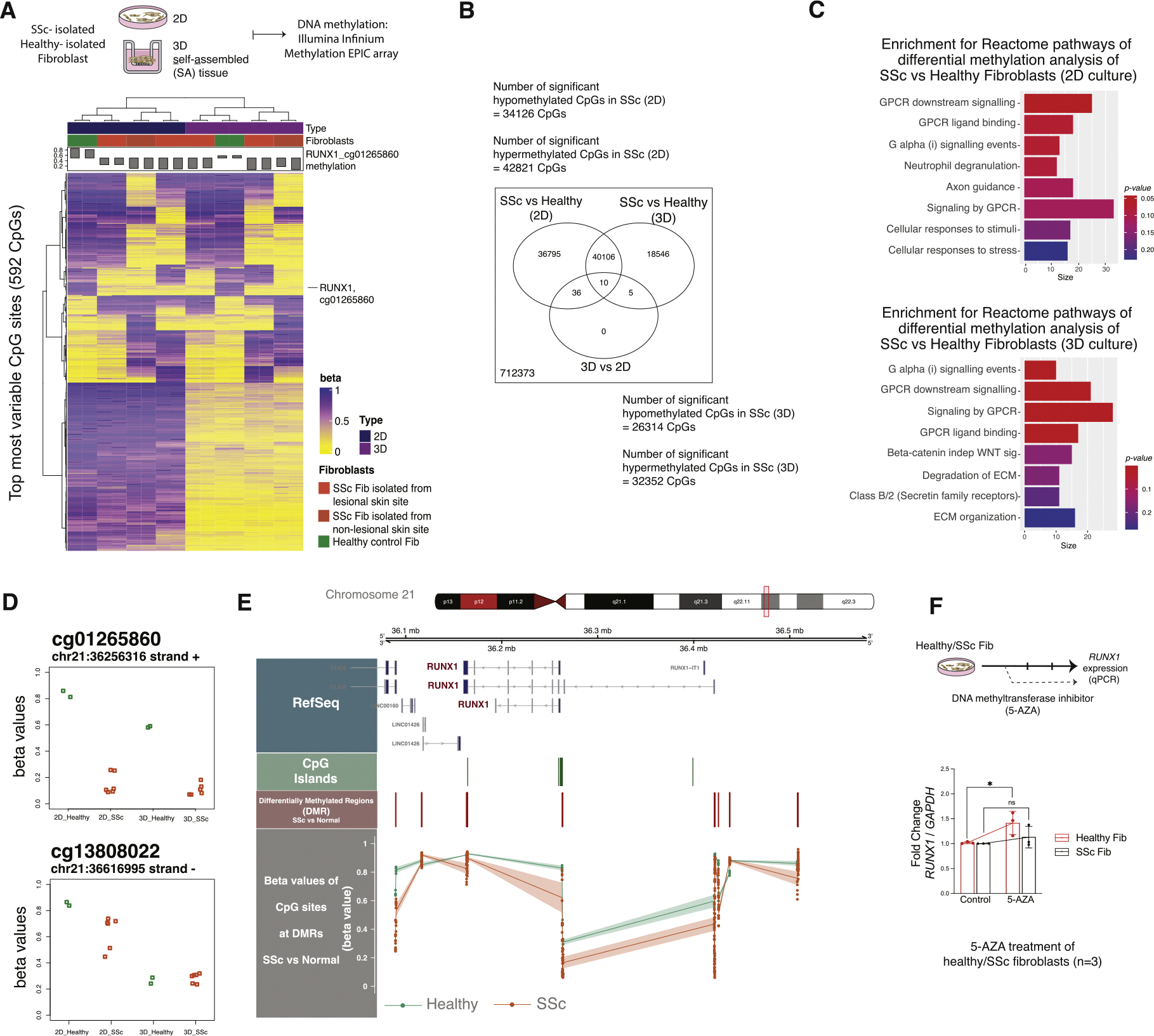
*RUNX1* is hypomethylated in SSc fibroblasts. (A) DNA methylation profile of 2D- and 3D-cultured fibroblasts isolated from patients with dcSSc or healthy donors, created using Illumina’s Infinium Methylation EPIC array. Heatmap shows top 592 methylated CpG sites, with blue/yellow gradient of beta values. The bar plot on top shows the *RUNX1* beta value that is labelled within the heatmap, showing that *RUNX1* is hypomethylated in dcSSc samples. (B) Result of paired-wise differentially methylated CpGs and the number of significant CpGs in each group. (C) Pathway enrichment analysis of Reactome gene sets using the top significant CpGs identified in (B) for each 2D and 3D culture. (D) The beta values of representative CpGs in *RUNX1* locus in 2D and 3D SSc and healthy conditions. (E) *RUNX1* locus on chromosome 21 and common CpG islands in green. The differentially methylated regions (DMRs) are identified between SSc and healthy samples are shown in red. The beta values corresponding to the CPGs at DMRs for SSc (in orange) and healthy (in green). (F) *RUNX1* expression rate in isolated healthy and SSc fibroblasts (n = 3) treated with DNA methyltransferase inhibitor, 5-Aza-2′-deoxycytidine (5-AZA) for 72 hours. dcSSc, diffuse cutaneous SSc; RUNX1, runt-related transcription factor 1; SSc, systemic sclerosis; 3D, 3-dimensional; 2D, 2-dimensional. G-Protein Coupled Receptor (GPCR ).

Samples clustered based on the most variable probes and were grouped primarily by the type of culture (ie, 2D or 3D) and then by the type of sample (ie, SSc or healthy) (Fig 4A). In 1 set of samples, differences were noted in several CpG sites when comparing fibroblasts from lesional vs nonlesional skin isolated from the same donor, suggesting that anatomic, site-specific differences in methylation patterns exist in isolated cells. To determine if the method of cell culture (2D vs 3D) significantly impacts the epigenetic landscape of SSc or healthy donor fibroblasts, we performed probe-wise differential methylation analysis between SSc and healthy samples in each culture type using the limma package. As shown in Figure 4B, 34,126 CpGs were found to be significantly hypomethylated in 2D cultured fibroblasts and 42,821 CpGs were hypermethylated in SSc samples relative to healthy controls. In 3D culture, 26,314 and 32,352 CpGs were hypomethylated and hypermethylated, respectively, in SSc samples compared with healthy controls. Among all significant CpGs (hypo- or hypermethylated), 40,106 were common between the 2D and 3D culture, which suggests that the culture system may affect cytosine methylation rates in fibroblasts (Fig 4B). However, it is possible that these differences in CpG methylation may not translate to altered gene regulation and pathway activity. Therefore, we used the top significant probes to analyse the enrichment of Reactome pathways using the methylGSA package, which accounts for the number of CpGs per gene. As shown in Figure 4C, there were significant pathway differences between SSc fibroblasts and healthy control cells in both 2D and 3D culture, while no significant pathway differences were identified as a result of differences in culturing conditions.

Significantly, analysis of the most highly variable CpG sites identified 1 CpG annotated at the *RUNX1* genomic locus (cg01265860, located on the positive/sense strand of ch21:36256316), ie, hypomethylated in all SSc fibroblasts compared to healthy control fibroblasts. Moreover, we also found additional probes—including cg09019048 and cg10011479 on the sense strand and cg13808022 on the antisense strand—that were located on the *RUNX1* locus and were also hypomethylated (Fig 4D, Supplementary Fig S5). Hypomethylation in a gene on the sense strand is often associated with increased gene expression. While methylation on the antisense strand may not directly impact the expression of the gene, it can play a regulatory role potentially through effects on RNA stability or processing.

Next, the differentially methylated regions (DMRs) between SSc and healthy fibroblasts were analysed to identify proximal CpGs that are concordantly differentially methylated (Supplementary Table S7). Figure 4E shows the *RUNX1* locus on chromosome 21 using the hg19 reference genome. The *RUNX1* gene is indicated and common CpG islands are shown (Fig 4E). Several DMRs were evident between SSc and healthy control fibroblasts, and analysis of the average beta values of CPGs at DMRs surrounding *RUNX1* demonstrated that the SSc samples were hypomethylated at almost all the sites compared to healthy controls (Fig 4E). For validation, we treated 3 healthy and 3 SSc-isolated fibroblasts with a DNA methyltransferase inhibitor, 5-Aza-2′-deoxycytidine (5-AZA). This commonly results in passive DNA demethylation over successive cell divisions and reactivation of epigenetically silenced gene. After 72 hours, we observed a significant upregulation of *RUNX1* expression in healthy fibroblasts (Fig 4F), suggesting that this gene is epigenetically repressed under normal conditions and can be reactivated upon DNA demethylation. There was no change in the gene expression in SSc fibroblasts consistent with the hypomethylated state of RUNX1 in these cells. Taken together, our data suggest that the significantly elevated *RUNX1* expression observed in patients with SSc is due to epigenetic dysregulation, and DNA methylation plays a regulatory role in controlling *RUNX1* expression in fibroblasts.

### RUNX1 is required for fibroblast activation, proliferation, and contraction

**Figure 5.**
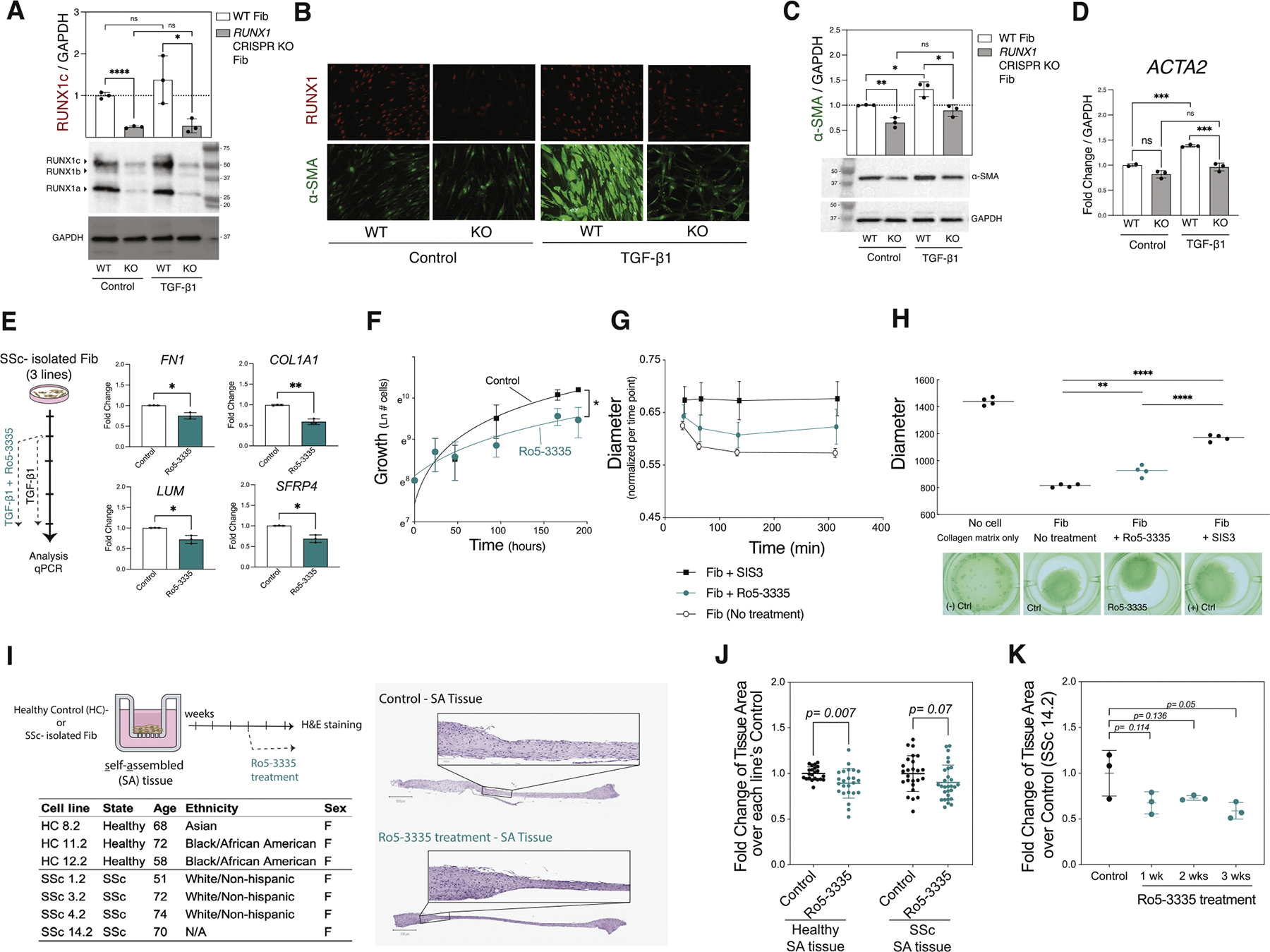
RUNX1 contributes to fibroblast activation, proliferation and contraction. (A) RUNX1 western blot of CRISPR-generated RUNX1 KO and wild-type (WT) fibroblasts under the TGF-*β*1 stimulation vs control. RUNX1 isoforms of a, b, and c were marked in the blot by arrows. (B) *α*-SMA and RUNX1 IF staining of KO and WT fibroblasts under the TGF-*β*1 stimulation vs control. (C) *α*-SMA western blot of KO and WT fibroblasts under the TGF-*β*1 stimulation vs control. (D) *ACTA2* mRNA expression of KO and WT fibroblasts under the TGF-*β*1 induction vs control. (E) Fold change expression of *FN1, COL1A1, LUM*, and *SFRP4* in TGF-*β*1-induced SSc fibroblasts treated with Ro5–3335 compared to control (3 lines of SSc fibroblasts, 2 replicates each). (F) Proliferation curve of normal human dermal (NHD) fibroblasts in the presence and absence of Ro5–3335. (G,H) The 3D collagen contraction assays, fixed (G) and floating (H) models, of NHD fibroblasts treated with Ro5–3335 (4 replicates for each condition). SIS3 (SMAD3 inhibitor) was used as positive control that significantly eliminates the contraction ability of fibroblasts. Negative control is collagen matrix with no fibroblasts. The overhead pictures represent 1 replicate for each condition. (I) 3D self-assembled (SA) tissue constructs from the healthy- and SSc-isolated fibroblast lines with donors’ clinical characteristics. H&E staining of representative untreated and Ro5–3335-treated tissues. (J) Tissue area fold change of each cell line over the control for healthy and SSc SA tissues. Data from 3 healthy and 4 SSc lines, 3 replicates per line, repeated in 2 independent sets. (K) Change in area of an SSc-isolated SA tissue when treated for 1, 2, or 3 weeks with Ro5–3335 compared to control (Student’s *t* test *P* value: **.001-.01, ****<.0001 in GraphPad Prism v9). *α*-SMA, alpha smooth muscle actin; H&E, haematoxylin and eosin; KO, knockout; RUNX1, runt-related transcription factor 1; SSc, systemic sclerosis; TGF-*β*, transforming growth factor-*β*;Clustered Regularly Interspaced Palindromic Repeats (CRISPR),Smad Family Member 3 (SMAD3),Glyceraldehyde-3-Phosphate Dehydrogenase (GAPDH), Not Applicable (N/A), Quantitative Polymerase Chain Reaction (QPCR), Quantitative Polymerase Chain Reaction, Immunofluorescenc (IF).

To determine the effect of RUNX1 deletion in fibroblasts, we generated the RUNX1 knockout (KO) in human dermal fibroblasts using a CRISPR/Cas9 (Associated Protein 9) system (Supplementary Fig S6). Validation of the KO clone was performed by sequencing and western blotting to confirm loss of RUNX1 protein. RUNX1 protein was eliminated in CRISPR-generated RUNX1 KO fibroblast and maintained at the same level under the TGF-*β*1-induced condition (Fig 5A). We evaluated the expression of *α*-SMA, a key marker of myofibroblast activation, using immunofluorescence staining. We observed a significant reduction in *α*-SMA expression in the *RUNX1* KO fibroblasts compared to wild-type. This decrease was further corroborated by quantitative measurements of *α*-SMA protein levels as well as *ACTA2* gene expression, both of which showed consistent downregulation in the KO cells. Importantly, this reduced expression of *α*-SMA protein and *ACTA2* mRNA was maintained even under stimulation with TGF-*β*1 (Fig 5B–D). These findings suggest that the *RUNX1* KO attenuates the fibrotic response mediated by TGF-*β*1.

The compound Ro5–3335 has been reported to directly interact with RUNX1 and its heterodimeric partner CBF*β*, repressing RUNX1/CBF*β*-dependent transactivation in reporter assays [38]. Therefore, we hypothesised that Ro5–3335 would attenuate SSc fibroblast activation and contraction by inhibiting RUNX1 activity. To test this, we treated 3 lines of TGF-*β*1-activated SSc fibroblasts with Ro5–3335. As shown in Figure 5E, the levels of ECM component genes such as *FN1, COL1A1, LUM,* and *SFRP4* were significantly reduced by 20 μM Ro5–3335. Other concentrations of Ro5–3335 were tested on several markers, confirming that the effect is concentration dependent (Supplementary Fig S7). Furthermore, Ro5–3335 reduced the proliferation rate of fibroblasts over the course of 3 days (Fig 5F). Inhibition of RUNX1 activity by Ro5–3335 also significantly reduced the ability of NHD fibroblasts to contract collagen gel matrices (Fig 5G,H).

Next, we assessed the effect of RUNX1 inhibition on dermal matrix formation using the 3D SA tissue model (Fig 5I). This *in vitro* SSc model recapitulates the fibrotic SSc phenotype present in the skin of patient with SSc as previously determined by histology and transcriptomics [36,37]. We observed that RUNX1 inhibitor, Ro5–3335, reduced the total area of tissues in healthy (n = 3) and most SSc lines (3 of 4 lines) (Fig 5J,K). Furthermore, time-course analysis demonstrated a sustained reduction in matrix formation after 1, 2, and 3 weeks of RUNX1 inhibition compared to the control (Fig 5G). Collectively, these results demonstrate that RUNX1 regulates key ECM gene expressions, fibroblast cell proliferation, matrix formation, and contractility in a collagen gel matrix and indicates an important role for RUNX1 as a driver of pathogenic SSc fibroblast activation.

## DISCUSSION

Efforts to fully characterise SSc dermal fibroblast heterogeneity and translational states are limited by tissue scarcity, anatomic site-specific differences, and a paucity of reliable fibroblast subset markers [39]. Defining SSc-specific fibroblast regulatory programmes that drive fibrosis, or conversely promote regeneration, is essential for achieving favourable healing outcomes. This work establishes *RUNX1* as a key player in the regulation of SSc fibroblast activation. We demonstrated that *RUNX1* is enriched in both the lesional and nonlesional skin of patient with SSc, with the highest expression in patients with dcSSc. Because *RUNX1* is aberrantly expressed in both lesional and nonlesional skin, *RUNX1* may be a driver of SSc pathogenesis in a prelesional state [32]. Additionally, higher expression of *RUNX1* is correlated with increased disease severity in patients with SSc, as evidenced by a greater risk for elevated mRSS and the presence of ILD.

Konkimalla et al [40] have recently shown that *RUNX1* is a key driver of alveolar fibroblast transitional states in multiple lung-injury mouse models, and that loss of *Runx1* decreases pulmonary levels of ECM components and their assembly. However, the mechanisms by which RUNX1 controls the expression of ECM components in TGF-*β*-activated fibroblasts are not yet clear. In this regard, TGF-*β* signalling is known to enhance fibrogenesis and to stimulate the production of ECM components, including collagen, fibrillin, laminin, fibronectin, proteoglycans, and elastin [41–43]. Previous studies have shown that RUNX1 is essential for mesenchymal stem cell proliferation and myofibroblast differentiation, and associates with the SMAD-dependent TGF-*β* signalling pathway to directly bind promoters and enhancers of multiple cell-state-specific genes related to cell proliferation and migration [9,44–46]. Consistent with these results, we demonstrated that the inhibition of RUNX1 via Ro5–3335 reduced dermal fibroblast proliferation, contraction, and 3D *in vitro* tissue matrix formation. Recognising that Ro5–3335 may have off-target effects, we used multiple methods to eliminate RUNX1 function. Collectively, these data provide additional support for RUNX1 as an integral driver of fibroblast activation in both 2D culture and a more biologically relevant 3D tissue culture.

Moreover, *RUNX1* upregulation is a hallmark of several other fibrotic diseases. For example, *RUNX1* is expressed in human retinal microvascular endothelial cells in proliferative vitreoretinopathy [47]. In agreement with our findings, inhibition of RUNX1 activity resulted in a significant reduction of retinal lesion size and reduced migration, proliferation, and tube formation of human retinal microvascular endothelial cells *in vitro* [47–49]. Overexpression of *RUNX1* is associated with enhanced liver fibrosis [50], and RUNX1 inhibition significantly mitigates pathology in models of pulmonary fibrosis [22]. RUNX1 binds to the transcriptional coactivator Yes-Associated Protein 1 (YAP), a key regulator in the Hippo signalling pathway, which has a role in differentiation and homeostasis of myofibroblast and the endothelial-to-mesenchymal-transitioning fibroblasts in SSc [51,52].

We have also demonstrated that *RUNX1* is hypomethylated in the SSc-derived fibroblast genome, indicating that the increased *RUNX1* expression observed in patients with SSc is likely due to dysregulation at the epigenetic level. 5-AZA treatment increased expression in healthy but not SSc fibroblasts consistent with these methylation results. Using single-cell multiomic analysis, we showed that *RUNX1* is enriched in a subpopulation of fibroblasts in an SSc-derived self-assembled skin-equivalent (saSE) tissue model at levels similar to those observed in SSc skin biopsies [53]. Using a single-cell sequencing assay for transposase-accessible chromatin (scATAC-seq), this study identified a fibroblast subpopulation with elevated TF motifs for *RUNX1* and *RUNX2*, together with *SMAD3, SMAD5*, and *NFKB1* [53]. This population was also characterised by increased expression of multiple collagens and profibrotic genes, including *POSTN* and *LUM* [53]. In light of DNA methylation and enriched chromatin accessibility data, which indicate higher TF motif accessibility, these observations imply that SSc fibroblasts are more susceptible to activation and differentiation under the influence of RUNX1.

Recent scRNA-seq from SSc skin biopsies identified distinct dermal SSc dermal fibroblast subsets [33,34,51,54]. An increased proliferation of *SFRP2*^*hi*^*PRSS23*^+^ fibroblasts, which represent a putative myofibroblast progenitor population, with *RUNX1*^*high*^ activity was identified in SSc skin [33]. Notably, dysregulation of associated genes in the *Wnt* signalling pathway, *SFRP2* and *SFRP4* (*Secreted Frizzled-Related Protein 2 and 4*), has been implicated in the pathogenesis of fibrosis disease [55]. Another scRNA-seq and scATAC-seq study identified a distinct SSc-specific subpopulation of fibroblasts termed as *LGR5*^+^-scleroderma-associated fibroblasts [34]. Our data demonstrated that *RUNX1*^*high*^ fibroblasts are *LGR5*^+^, suggesting that *RUNX1* is an SSc fibroblast TF that contributes to the altered phenotype and function of these cells. Another study by Ma et al [51] identified 2 major clusters of *SFRP*^+^ fibroblasts and *COL8A1*^+^ fibroblasts. *COL8A1*^+^ fibroblasts were located in the deeper dermis of SSc skin and expressed high levels of *ACTA2, SFRP4, POSTN*, and *PRSS23*, and also had the highest ECM score. We show here that *RUNX1* is activated in *COL8A1*^+^ cells. Regardless of the variation among the fibroblast subpopulations reported in the different cohorts, *RUNX1* expression is consistently linked to either the myofibroblast or SSc-specific fibroblast or progenitor population.

This study combines human skin data from multiple platforms and cohorts, ranging from bulk microarray to scRNA-seq, to validate of our findings at different levels of resolution. Leveraging gene expression, DNA methylation, and single-cell data of SSc fibroblasts, we discovered unique associations between disease-specific subpopulations of fibroblasts and *RUNX1*, which may serve as a potential novel therapeutic target for SSc. As *RUNX1* activity is tissue specific, our study is limited by the identification of the direct target genes in SSc fibroblasts. A limitation of our study is the use of only 2 to 3 biological replicates in the inhibition assays. Given the significant heterogeneity of RUNX1 expression observed across patients, a larger sample size is needed to capture the full biological variability. Additionally, our work in part relied on Ro5–3335, a compound with low potency and specificity; as such, results obtained with this inhibitor cannot be directly extrapolated to the clinical setting. Continued development of next-generation RUNX1 inhibitors with improved pharmacological properties is essential. Finally, future research should incorporate more advanced preclinical models, including *in vivo*, and patient-derived systems to thoroughly investigate RUNX1’s role and assess whether the inhibition of RUNX1 is sufficient to block disease progression and reverse established dermal fibrosis.

## Supplementary Material

Supp_material

Supp_table

Supplementary material associated with this article can be found in the online version at doi:10.1016/j.ard.2025.10.033.

## Data Availability

DNA methylation data have been deposited into the National Center for Biotechnology Information (NCBI) Gene Expression Omnibus (GEO) at Accession Number GSE311858. Single cell data for RUNX1 siRNA knockdowns have been deposited under Accession Number GSE312591.

## WHAT IS ALREADY KNOWN ON THIS TOPIC

- Dysregulation of dermal fibroblast activity leads to excessive collagen production and deposition, causing scar tissue formation and fibrotic lesions in systemic sclerosis (SSc).
- Transcription factors play a key role in driving functional diversity among dermal fibroblasts in SSc, and understanding their dysregulation in SSc fibrosis is critical for the development of new therapeutic strategies.
- Runt-related transcription factor 1 (RUNX1) is a member of the core-binding factor family that regulates proliferation, differentiation, and cell survival in multiple cell lineages.
- Current understanding of the role of RUNX1 in dermal fibroblasts and its extent of involvement in SSc dermal fibrosis is very limited.

## WHAT THIS STUDY ADDS

- Our observations indicate a potential association between the severity of dermal fibrosis and *RUNX1* expression levels in SSc skin.
- *RUNX1* is enriched in SSc-specific fibroblast subpopulations.
- We identify hypomethylated CpG sites near the *RUNX1* gene, which suggests their potential involvement in the upregulation of *RUNX1* in SSc dermal fibroblasts.
- Inhibition of RUNX1 activity by Ro5–3335, knockdown by siRNA, and RUNX1 knockout resulted in statistically significant changes in extracellular matrix components.
- Inhibition of RUNX1 activity caused a reduction in fibroblast contraction and proliferation rates.

## HOW THIS STUDY MIGHT AFFECT RESEARCH, PRACTICE OR POLICY

- The discovery of unique associations between disease-specific fibroblast subpopulations and RUNX1 provides the foundation for therapeutic targeting of RUNX1 in SSc.
