# Supplementary material for: RUNX1 is expressed in a subpopulation of dermal fibroblasts and is associated with disease severity of systemic sclerosis
Source: Ann Rheum Dis. Author manuscript; Available in PMC 2026 Mar 9. (PMC12969718; doi:10.1016/j.ard.2025.10.033)
Supplement: Supp_material [file NIHMS2148908-supplement-Supp_material.docx]

**Supplementary Data**

**Methods**

**DNA microarray data processing:**

***Agilent DNA microarray processing.*** Raw gene expression data from skin biopsies of patients with SSc and healthy controls were obtained from the gene expression omnibus (GEO) including: Hinchcliff *et al.*, 2013 (1) (GSE59787, Figure 1), Milano *et al.*, 2008 (2) (GSE9285, Supplemental Figure 2A), Pendergrass *et al.*, 2012 (3) (GSE32413, Supplemental Figure 2B), Franks *et al.*, 2019 (4) (GSE125362, Supplemental Figure 2C), Gordon *et al.*, 2018 (5) (GSE97248, Supplemental Figure 2D), and one database with TGF-β1-induced fibroblasts, Sargent *et al*., 2010 (6) (GSE12493, Figure 2). Information on all of the datasets is provided in Supplementary Table S1. The clinical data were either publicly available or collected upon request from the authors.

Samples were assayed using one of the Agilent Technologies 2-channel DNA microarrays platforms: Agilent-014850 Whole Human Genome Microarray 4x44K G4112F platform, Agilent-012391 Whole Human Genome Oligo Microarray G4112A platform, or Agilent-028004 SurePrint G3 Human Gene Expression 8x60K Microarray platform. Raw data in the format of the .gpr file generated by GenePix scanner were processed through Bioconductor ‘*limma’* package by reading the Cy3/green channel of the arrays (mean foreground and median background signal intensities). Low-quality spots were weighted, and outlier spots were identified using median absolute deviation (~0.6% spots were found to be outliers based on the quality). The method of normexp (7) was used for background correction followed by quantile normalization to enable cross-array comparisons. Probes with >80% of 1.3-fold or lower intensity over local background were considered very-low-expressed and were removed. The Agilent’s annotations were downloaded from “Agilent Technology eArray” webpage for each of the microarray platforms. Using these annotations, probe IDs were then converted to gene symbols and ‘*collapseRows’* (8) was used to collapse the normalized log2 intensities based on gene symbols. Finally, the normalized log2 expression matrices were used to measure the expression rate of *RUNX1* in all arrays and time points.

The Hinchcliff *et al.*, 2013 (GSE59787) dataset includes skin biopsies from longitudinal data with samples from baseline and at 6, 12, 24, and 36 months from both lesional and non-lesional skin. In all databases, batches were evaluated before the analysis and corrected. Patients who had been diagnosed with the disease within 2 years of the date of skin biopsy were assigned as “early stage” and the rest were “late stage”. For Pendergrass *et al.*, 2012 (GSE32413) only samples from one batch at baseline were used. Gordon *et al.*, 2018 (GSE97248) had samples from baseline and 12 months; therefore, both time points were analyzed. In the TGF-β1-induced fibroblast (GSE12493) dataset, two separate platforms were used (Agilent-014850 and Agilent-012391). As a result, data were first analyzed separately under each platform and then carefully merged by ‘ComBat’ from ‘sva’ package (v. 3.46.0) with a design matrix to control for biological differences between clinical conditions while performing batch effect correction.

***Illumina DNA microarray processing.*** Raw microarray data were obtained from GEO using accession ID GSE58095 (Assassi *et al.*, 2015) (9). The data were generated on the Illumina HumanHT-12 V4.0 expression beadchip platform and processed using the ‘lumi’ package (v. 2.50.0) in R (v. 4.2.2). Probes with high detection (*P*-values > 0.01) were removed to filter out probes with low signal-to-noise. The ‘arrayQualityMetrics’ package (v. 3.54.0) was utilized to perform quality control assessment of the processed data. The ‘illuminaHumanv4’ was employed to annotate the probes. This package provides annotation information for probes, including gene symbols and genomic coordinates. Normalized and transformed expression values were then obtained using quantile method by ‘*normaliseIllumina’* implemented in the ‘*beadarray’* package. Similar to the Agilent pipeline, we used ‘*collapseRows’* to collapse the intensities based on gene symbol.

***Affymetrix DNA microarray processing.*** Raw microarray data were obtained from GEO using accession ID GSE55036 (Rice *et al*., 2015) (10). The data were Affymetrix DNA microarrays that were processed using the ‘Affy’ package (v. 1.76.0). The ‘gcrma’ algorithm was utilized to convert the raw data into an ExpressionSet object. This algorithm employs robust multi-array averaging (RMA) with additional sequence-based correction to estimate expression values. Quality assessment was performed using the Relative Log Expression (RLE) and Normalized Unscaled Standard Error (NUSE) metrics. These metrics assess the distribution of expression values across samples and provide insights into the overall data quality and consistency. The ‘*arrayQualityMetrics*’ package was used to assess various aspects of microarray data quality. Based on these QC assessments, three low-quality arrays from patient 19 (at 3, 7, and 24 weeks) were removed from subsequent analysis. The annotation package ‘*hgu133a2.db’* (v. 3.13.0) and ‘*collapseRows’* were utilized to annotate and convert the probes to gene symbols.

**Differential expression analysis (DEG):**

Differential expression analysis was performed on the processed microarray data using the ‘*limma*’ package. Contrast of interest between 12 hours after exposure and baseline was specified using a contrast matrix. Significant DEGs were annotated and further analyzed to interpret biological significance using Reactome gene sets.

**Gene Set Variation Analysis (GSVA):**

The resultant normalized log2 expression matrices of GSE59787 and GSE55036 were subsequently employed in Gene Set Variation Analysis (GSVA). GSVA is a non-parametric method for estimating variations in gene set enrichment within a given set of samples derived from an expression dataset (11). The ‘*gsva*’ (v. 1.51) algorithm was used to calculate the samples’ enrichment scores for cell-type signatures across all samples. The gene sets are listed in Supplemental Table S2.

**RUNX1 Knockdown with siRNA and single cell RNA sequencing:**

Skin fibroblasts isolated from SSc patients (n=2) were treated for 72 hours with either RUNX1 siRNA (Dharmacon, catalog ID E-003926-00-0010) or non-targeting control pool siRNA (NC, Dharmacon, D-001910-10-05). Cells were grown in RPMI-1640 media supplemented with 10% FBS and 1% Penicillin/ Streptomycin. Cells were cultured at 37°C in a humidified incubator with of 5% CO2 and maintained by sub-culturing twice a week. A day prior to treatment, 7.5 x10^4^ cells were seeded into each well of a 6-well plate in 5 ml complete tissue culture media. For treatment, cells were incubated with RUNX1 siRNA or NC (1µM each in 1.3 ml Accell siRNA delivery media) for 72 hours, after which they were detached with 0.25% Trypsin-EDTA and centrifuged.

**scRNA-Seq library preparation, sequencing, and alignment:**

Cell pellet was resuspended in 1X PBS+0.04% BSA, and cell viability was assessed with 0.4% Trypan Blue. Cells were fixed and permeabilized with 10X Genomics fix/perm buffer for 16 hours at 4°C, followed by incubation with quenching and enhancer buffer on ice, as per the instructions in Chromium Next GEM Single Cell Fixed RNA Sample Preparation Kit (10X Genomics, catalog number PN-1000414). Samples were stored in 10% glycerol until sequencing. Sequencing was performed at the University of Michigan Advanced Genomics Core. Data processing including quality control, read alignment (hg38), and gene quantification was conducted using the 10X Cell Ranger software. The samples were then merged into a single expression matrix using the Cell Ranger aggr pipeline.

**Processing of skin single-cell RNA sequencing:**

Single-cell RNA-seq data from Tabib *et al.* was downloaded from GEO: GSE138669. Raw sequencing data were initially assessed through *Cell Ranger*, v. 6.0.1. The High-Performance Computing (HPC) resources at Dartmouth College were used to integrate the data. For data processing, the ‘*Seurat*’ package v. 4.4.0 was used. Quality control steps were implemented to filter cells, leaving a dataset with unique feature counts between 200–2,500, less than 5% mitochondrial reads, and no doublets. Data was of low-read depth (majority of cells <50 features per cell), so a large number of cells were lost through filtering steps. Our quality control filtering resulted in a total of 33,171 single-cell transcriptomes.

**single cell RNA-seq data analysis:**

The count matrix was normalized using the ‘*NormalizeData*’ method implemented in ‘Seurat’, then gene expression values were scaled across cells, using the ‘*ScaleData*’ function. Normalized and scaled expression values were then used for downstream analyses. Cell type was assigned to each cluster using gene expression distribution of cell-type-specific markers shown in each original publication and using ‘*FindNeighbors*’ and ‘*FindClusters*’ functions.

To identify fibroblast clusters based on similar gene expression profiles, the ‘*FindClusters*’ were applied only to fibroblast clusters using a graph-based clustering approach. A total of 10 clusters were found, consistent with the original publication.

To calculate the total *RUNX1* expression on each sample, ‘*AggregateExpression*’ was performed on the log normalized data. The library of ‘*scCustomize*’ was used to make UMAP plots, feature plots, and density plots. The ‘*FindMarkers*’ function was used to identify the top genes between *RUNX1^high^* and *RUNX1^low^* fibroblast populations.

To calculate the cluster composition based on condition (siRUNX1 and siNC), the number of cells for each condition from each cluster was counted. The counts were then divided by the total number of cells for each condition and scaled to 100 percent for each cell type. Differential expression analysis between siRUNX1 and siNC was carried out using the *FindMarkers* function, and the top pathways were selected with the genes adjusted p-value less than 0.05 by *enricher* function from *clusterProfiler* package.

**Fibroblast isolation and culture:**

SSc and healthy dermal fibroblast lines were isolated and expanded from skin punch biopsies as described previously (12–14). Isolated fibroblasts were stored in liquid nitrogen in stocks of 0.5 million cells in freezer media [70% fibroblast media, 20% FBS (fetal bovine serum, HighClone), and 10% dimethyl sulfoxide (DMSO)]. Fibroblasts were maintained in complete growth media [90% DMEM (Dulbecco’s Modified Eagle Media), 10% FBS, 1% HEPES (Millipore Sigma, Germany)], and 1% penicillin-streptomycin (Corning). Fibroblasts at passages less than 8 were used to seed for 2D and 3D tissue cultures. A summary of demographic and clinical characteristic of the patients and healthy controls who donated skin biopsies for DNA methylation profiling is provided in Supplemental Table S4. For the RUNX1 inhibition assay, TGF-β1-induced (10 ng/mL) (R&D systems, #240-B) SSc fibroblasts (n=3) were treated with 20 μM Ro5-3335 (TOCRIS, #4694). For demethylation assay, healthy and SSc fibroblasts (n=3) were treated with 1 μM 5-AZA for 72 hours.

**Generation of RUNX1 Knockout Human Dermal Fibroblasts Using CRISPR/Cas9:**

The RUNX1 knockout (KO) in human dermal fibroblasts was generated using a CRISPR/Cas9 system. The sgRNA sequence targeting RUNX1 (TGGAAGGCGGCGTGAAGCGG) was designed using the Synthego Knockout Guide Design web interface, and complementary synthetic oligonucleotides (RUNX1SGRNA1F1: CACCGTGGAAGGCGGCGTGAAGCGG; RUNX1SGRNA1R1: AAACCCGCTTCACGCCGCCTTCCAC) were purchased from Millipore-Sigma, annealed, and ligated into the BbsI site of the pSpCas9(BB)-2A-GFP (PX458) vector (Addgene # 48138) following our established protocols (15). Ligated plasmid was transformed into competent E. coli (ThermoFisher # C737303), and positive clones were selected, purified (Qiagen # 27106), and verified by sanger sequencing. The verified plasmid was transfected into human dermal fibroblasts using TransfeX (ATCC # ACS4005). After 24 hours of transfection, GFP-positive cells were single-cell sorted at University of Michigan Flow Cytometry Core into 96-well plates, expanded, and subsequently re-plated into 12-well plates. Genomic DNA from these clones was extracted and the region surrounding the CRISPR target was PCR-amplified (RUNX1-PCR-F1: GGGTCCTAACTCAATCGGCT; RUNX1-PCR-R1: CAGAGGAAGTTGGGGCTGTC); Sanger sequencing was used to identify clones with homozygous mutations. Final validation of knockout clones was performed by western blotting (Abcam # ab240639) to confirm loss of RUNX1 protein.

**Immunofluorescence, Western Blot, and qPCR Assessment of RUNX1 and αSMA in WT and RUNX1 KO Fibroblasts With and Without TGF-β1 Induction:**

Wild-type (WT) and RUNX1 knockout (KO) fibroblasts were cultured in 6-well plates for quantitative PCR (qPCR) and western blot experiments, and on glass coverslips (in 6 well plate) for immunofluorescence (IF) staining. For western blot and qPCR, cells were treated with recombinant TGF-β1 (10 ng/mL, R&D Systems Cat # 240-B-010/CF) for 72 hours. For IF staining, cells on coverslips were treated with TGF-β1 for 24 hours.

**Quantitative real-time PCR:**

Total RNA was isolated using the RNeasy Fibrous Tissue Mini Kit for 3D tissues and RNeasy Plus Mini Kit for 2D cultures (Qiagen, #74704 or #74136) per manufacturer’s instructions. Complementary DNA (cDNA) was synthesized from 100 ng total RNA using the qScript™ Ultra cDNA SuperMix (QuantaBio, #95161). Quantitative real-time PCR was performed using TaqMan Universal PCR Master Mix (Life Technologies, #4324020) for human *GAPDH, RUNX1, COL1A1, FN1, LUM, ACTA2, SFRP4* (Applied Biosystems, Hs02786624_g1, Hs01021970_m1, Hs00164004_m1, Hs01549976_m1, Hs00929860_m1, Hs00426835_g1, Hs00180066_m1). The StepOnePlus Real-Time PCR System (Applied Biosystems) was used and threshold cycle (Ct) values were determined using Opticon software. Relative gene expression levels were analyzed using the 2^–ΔΔCt method, with *GAPDH* used as the internal control for normalization. Fold changes in gene expression were calculated relative to the control.

**Western blotting:**

Fibroblasts were harvested and lysed in Pierce RIPA buffer (Thermo Fisher # AAJ63306AK or Thermo Fisher # 89900) supplemented with protease and phosphatase inhibitor cocktails (PIA32961). Protein concentration was determined using the BCA Protein Assay Kit (Pierce). Equal amounts of total protein were denatured by boiling in Laemmli sample buffer containing 5% β-mercaptoethanol for 5 minutes and separated by SDS-PAGE on 8–12% polyacrylamide gels 4–15% Mini-PROTEAN® TGX™ Precast protein gels. Proteins were transferred onto PVDF membranes (Millipore) using a wet transfer system at 100 V for 1 hour.

Membranes were blocked with the mixture of 2.5% non-fat dry milk plus 2.5% BSA in Tris-buffered saline (Bio-Rad) with 0.1% Tween-20 (TBS-T) and then probed with primary antibodies against the proteins RUNX1 (PAJ-19638), alph-smooth muscle actin (Abcam, ab5694), and polyclonal GAPDH (PA1-987), followed by secondary antibody (goat anti-rabbit IgG, Abcam ab6721). After washing three times with TBS-T, membranes were incubated with HRP-conjugated secondary antibodies for 1 hour at room temperature. Following three additional washes, signal detection was performed using enhanced chemiluminescence (ECL) reagents SuperSignal™ West Femto Maximum Sensitivity Substrate or Pierce™ ECL Western Blotting Substrate (Thermo Scientific) and visualized on a chemiluminescence imaging of Bio-Rad ChemiDoc. Band intensities were quantified using Image Lab software and normalized to loading controls of GAPDH.

**Immunofluorescence (IF):**

For IF staining, coverslips were washed 3X in 1X PBS following 1-2 weeks in 4C preserved in 0.02% sodium azide-PBS solution. The coverslips were blocked in the wells with 0.5mL 4% FBS-PBS for 1 hour at room temperature gently rocking, then blocking liquid was aspirated off. A combination primary antibody dilution was made in 4% FBS-PBS to final concentrations of 1:200 anti-RUNX1 (Abcam ab240639) and 1:500 anti-alpha smooth muscle actin (Abcam ab7817) and then 0.5mL of antibody dilution was added to each coverslip in wells. Primary antibodies were incubated O/N (16-24 hours) at 4C gently rocking. The following day, the antibody liquid was aspirated off followed by 3X washes in 1X PBS, 5 minutes each at room temperature. A combination secondary antibody dilution was made with 1:100 Donkey anti-rabbit TRITC (Thermo Fisher A16040) and 1:100 donkey anti-mouse AF488 (Thermo Fisher A21202) in 4% FBS-PBS and then added 0.5mL/well. Secondary antibodies were incubated for 1 hour room temperature rocking and protected from light, followed by 3X 5-minute room temperature washes in PBS, protected from light. Coverslips were retrieved from wells using forceps and pipette tip, dabbed on a Kimwipe to remove excess liquid, and inverted onto slides with 20uL ProLong Gold antifade reagent with DAPI (Invitrogen P36931). The coverslips with cells face-down to the slides were cured for 24 hours at room temperature protected from light and then used in imaging.

**Collagen contraction assay:**

Measurements of 3D fibroblast collagen contraction were performed as previously described (16). Briefly, culture plates are pre-coated with BSA and then 4x10^4^ fibroblasts/mL suspension in MCDB medium (Sigma, #M6395) mixed with collagen solution, which is a mix of one part collagen solution (Advanced BioMatrix, 6 mg/mL), one-part HEPES (pH 8), and two parts MCDB 2X. Then, 1 mL of media is added to the pre-coated wells and allowed to polymerize. The final collagen concentration is 1.2 mg/mL with 80,000 cells/mL. Cells were then incubated at 37°C with 5% CO2. For fixed contraction assays, cells were detached from the wells 48 hours post-polymerization and contraction was quantified by a decrease in gel diameter within 5 hours. For floating assay, the cells were detached from the plate immediately after polymerization and the gel diameter was measured after 48 hours. Where drug treatment was added, cells were pre-incubated with Ro5-3335 or SIS3 (TOCRIS, #5291, as positive control) for one hour prior to the assay. In principle, contraction in the floating model occurs in the absence of external mechanical load, whereas, in the fixed model, the attachment of the cell-collagen mixture to the tissue culture dish induces more stress fibers.

**Skin Tissue Culture:**

The 3D self-assembled (SA) tissues were grown as described previously (Cite). Briefly, 16,000 fibroblasts per insert seeded in Costar 0.4μm Transwell® plates (REF3413) or Millicell plates (for DNA methylation). Tissues were fed with fresh media twice a week at regular three to four-day intervals. The media consisting of a 3:1 ratio of DMEM/F12 (ThermoFisher Scientific) with the following supplements: 5% FCII (Hyclone), 8 mM HEPES (Sigma), 0.18 mM adenine (Sigma), 1 nM cholera toxin (Sigma), 10ng/ml EGF (PeproTech), 0.5 μg/ml hydrocortisone (Sigma), 5 μg/mL insulin (ThermoFisher Scientific), and 10 μg/ml of L-ascorbic acid-2-phosphate (Sigma), 10 mg/ml Ascorbic acid (Sigma-Aldrich). Ro5-3335 solution was added to the growth media at the end of week 3 and maintained for about 14 days.

At week five, three SA tissues were harvested from each line and paraffin embedded. The tissue was sectioned perpendicular to the cassette to obtain a 4 μm cross-section from the central region. The resulting sections were affixed to slides and stained with Hematoxylin and Eosin (H&E) using the Tissue-Tek Prisma Stainer (Sakura Finetek USA) and the automated H&E staining protocol. Thickness of the tissue was determined using representative images of each tissue and the open-source software QuPath (version 0.2.3). The areas were calculated by running a script to automated measure area across all images as it is described by QuPath (17).

**DNA extraction, bisulfite conversion, and DNA methylation array:**

A total of 16 samples [eight samples from 2D monolayer cultures of fibroblasts isolated from patients with SSc or healthy controls, and eight samples from 3D cultures (2 replicates from each biological sample)] were selected for DNA extraction. Genomic DNA was extracted using the Qiagen QIAcube by QIAamp DNA Mini Kit (Hilden, Germany). To ensure that all samples met DNA quality control criterion, we used Agilent 4200 TapeStation (Genomic DNA ScreenTape, Agilent technologies, Santa Clara, CA). Between 500 ng and 1 µg of DNA from these 2D and 3D samples were bisulfite converted and processed according to the Illumina Infinium MethylationEPIC array protocols at the University of Southern California’s Molecular Genomics Core Laboratory (Los Angeles, CA).

**DNA methylation data processing:**

The raw intensity data (IDAT files) from the MethylationEPIC array were processed using the *‘minfi’* package (v. 1.44.0), which provides functions for reading, preprocessing, and analyzing DNA methylation data. We filtered the probes with *detection* *P-values* < 10^-5^, which compares the total, methylated (M), and unmethylated (UM) signals for each probe to the background signal level. Thereby, probes with high *detection P-values* or high background noise were excluded from further analysis. The data were then normalized using quantile normalization.

Probes with CpGs at common single nucleotide polymorphisms (SNPs) or that tracked to sex chromosomes were filtered out, leaving a total of 807,871 probes. The ‘*Illumina Human Methylation EPICanno.ilm10b4.hg19’* (v. 0.6.0) package was used to annotate the CpG probes. This package provides annotation information for probes, including corresponding gene reference, genomic coordinates, type of strand, and probe sequence. Finally, methylation levels were calculated through both beta values (β=M/(M+UM)) and M-values (M-value=log2(M/UM)), which were used for downstream analyses. Topmost variable probes were used for unsupervised hierarchical clustering to examine how the samples cluster with each other using beta values. Additionally, probe-wise differential methylation analyses were performed using the ‘*limma’* package on M-values with a design matrix accounting for SSc vs. healthy conditions in 2D and 3D culture systems. The most significant differentially methylated CpGs were subsequently used for gene set enrichment analysis by the ‘*methyglm’* function from the ‘*methyGSA’* package; this carries out gene set analysis adjusted for the number of CpGs per gene.

**Differential methylation analysis of regions (DMRs):**

The ‘*DMRcate*’ package was used to identify differentially methylated regions (DMRs) between SSc and healthy samples using M-values, which are proximal CpGs of genes that are concordantly differentially methylated between the conditions. The DMRs corresponding to the *RUNX1* gene are provided in Supplemental Table S7.

**Statistical analyses:**

Statistical significance was determined using a Student’s *t-test*, paired *t-test*, or Pearson correlation test, as appropriate, and a *P*-value of <0.05 was considered significant. All data were analyzed using R (v. 4.2.2). For gene sets, effect size measurements of Hedge’s g were used to quantify the standardized mean difference between two groups for each gene set. Hedge’s g effect size is a measure of association and not a measure of statistical significance.

**Patient and Public involvement:**

Patients and/or the public were not involved in the design, or conduct, or reporting, or dissemination plans of our research.

**Ethical Approval**

The study was approved by the Committee for the Protection of Human Subjects at Dartmouth College (STUDY00016631), the Institutional Review Board (IRB) at Tufts Medical Center (12773: Complex 3-Dimensional in Vitro Human Skin Models for Scleroderma, 01535: Functional analysis of cellular diversity and cell-cell interactions in Scleroderma 3D skin-like tissues), and IRB at University of Michigan (HUM00065044). All participants provided written informed consent.

**Supplementary References:**

1. Hinchcliff M, Huang CC, Wood TA, et al. Molecular signatures in skin associated with clinical improvement during mycophenolate treatment in systemic sclerosis. *J Invest Dermatol*. 2013;133(8):1979-1989. doi:10.1038/jid.2013.130

2. Milano A, Pendergrass SA, Sargent JL, et al. Molecular subsets in the gene expression signatures of scleroderma skin. *PloS One*. 2008;3(7):e2696. doi:10.1371/journal.pone.0002696

3. Pendergrass SA, Lemaire R, Francis IP, Mahoney JM, Lafyatis R, Whitfield ML. Intrinsic gene expression subsets of diffuse cutaneous systemic sclerosis are stable in serial skin biopsies. *J Invest Dermatol*. 2012;132(5):1363-1373. doi:10.1038/jid.2011.472

4. Franks JM. *Machine Learning Approaches for Patient Stratification and Precision Medicine in Systemic Sclerosis*. Ph.D. Dartmouth College; 2019. Accessed October 21, 2021. https://www.proquest.com/pqdtlocal1006600/docview/2232683551/abstract/FFCF36B07AE94A61PQ/3

5. Gordon JK, Martyanov V, Franks JM, et al. Belimumab for the Treatment of Early Diffuse Systemic Sclerosis: Results of a Randomized, Double-Blind, Placebo-Controlled, Pilot Trial. *Arthritis Rheumatol Hoboken NJ*. 2018;70(2):308-316. doi:10.1002/art.40358

6. Sargent JL, Milano A, Bhattacharyya S, et al. A TGFbeta-responsive gene signature is associated with a subset of diffuse scleroderma with increased disease severity. *J Invest Dermatol*. 2010;130(3):694-705. doi:10.1038/jid.2009.318

7. Ritchie ME, Silver J, Oshlack A, et al. A comparison of background correction methods for two-colour microarrays. *Bioinforma Oxf Engl*. 2007;23(20):2700-2707. doi:10.1093/bioinformatics/btm412

8. Miller JA, Cai C, Langfelder P, et al. Strategies for aggregating gene expression data: the collapseRows R function. *BMC Bioinformatics*. 2011;12:322. doi:10.1186/1471-2105-12-322

9. Assassi S, Swindell WR, Wu M, et al. Dissecting the heterogeneity of skin gene expression patterns in systemic sclerosis. *Arthritis Rheumatol Hoboken NJ*. 2015;67(11):3016-3026. doi:10.1002/art.39289

10. Rice LM, Ziemek J, Stratton EA, et al. A longitudinal biomarker for the extent of skin disease in patients with diffuse cutaneous systemic sclerosis. *Arthritis Rheumatol Hoboken NJ*. 2015;67(11):3004-3015. doi:10.1002/art.39287

11. Hänzelmann S, Castelo R, Guinney J. GSVA: gene set variation analysis for microarray and RNA-seq data. *BMC Bioinformatics*. 2013;14:7. doi:10.1186/1471-2105-14-7

12. Huang M, Smith A, Watson M, et al. Self-assembled human skin equivalents model macrophage activation of cutaneous fibrogenesis in systemic sclerosis. *Arthritis Rheumatol*. n/a(n/a). doi:10.1002/art.42097

13. Huang M, Liu Z, Baugh L, et al. Lysyl oxidase enzymes mediate TGF-β1-induced fibrotic phenotypes in human skin-like tissues. *Lab Investig J Tech Methods Pathol*. 2019;99(4):514-527. doi:10.1038/s41374-018-0159-8

14. Huang M, Cai G, Baugh LM, et al. Systemic Sclerosis Dermal Fibroblasts Induce Cutaneous Fibrosis Through Lysyl Oxidase–like 4: New Evidence From Three-Dimensional Skin-like Tissues. *Arthritis Rheumatol*. 2020;72(5):791-801. doi:10.1002/art.41163

15. Sarkar MK, Uppala R, Zeng C, et al. Keratinocytes sense and eliminate CRISPR DNA through STING/IFN-κ activation and APOBEC3G induction. *J Clin Invest*. 2023;133(9):e159393. doi:10.1172/JCI159393

16. Shi-wen X, Eastwood M, Stratton RJ, Denton CP, Leask A, Abraham DJ. Rosiglitazone alleviates the persistent fibrotic phenotype of lesional skin scleroderma fibroblasts. *Rheumatol Oxf Engl*. 2010;49(2):259-263. doi:10.1093/rheumatology/kep371

17. Bankhead P, Loughrey MB, Fernández JA, et al. QuPath: Open source software for digital pathology image analysis. *Sci Rep*. 2017;7(1):16878. doi:10.1038/s41598-017-17204-5

**Supplementary Figures:**

**Supplemental Figure 1.** *RUNX1* expression rate for healthy and SSc patients at early or late stages of disease at baseline.


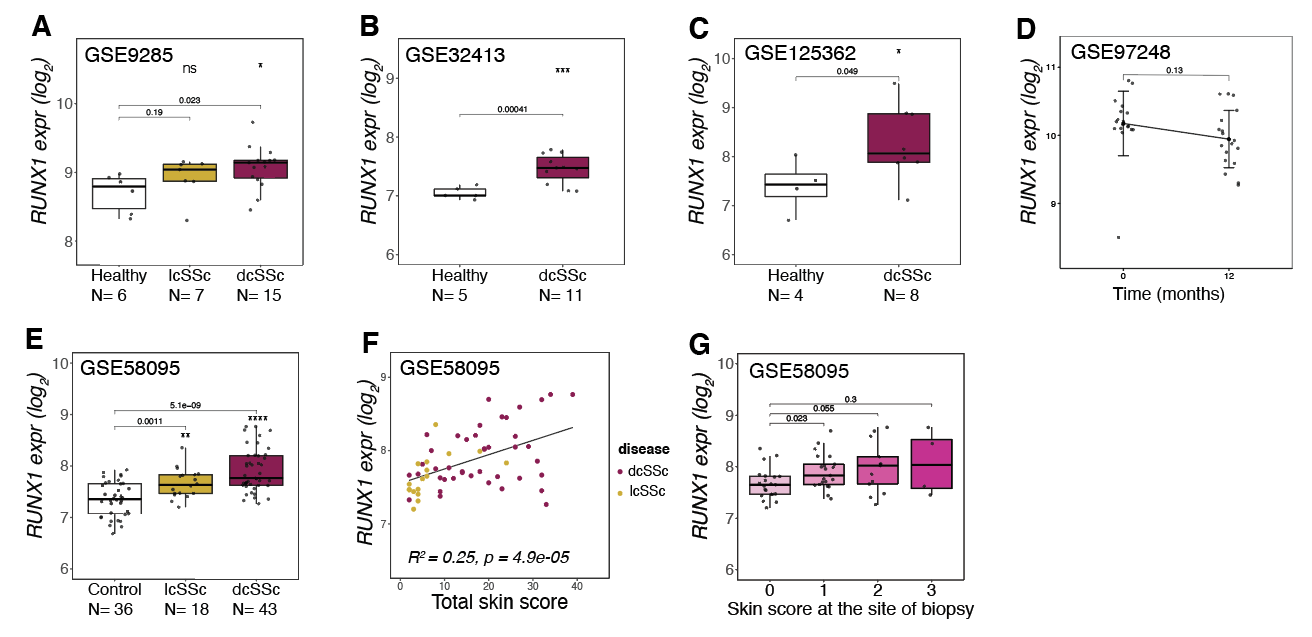


**Supplemental Figure 2.** (A) *RUNX1* expression rate in skin biopsies of dcSSc patients (*N*=15), lcSSc patients (*N*=7), and healthy donors (*N*=6) from the Milano *et al.*, 2008 study (GSE9285). (B) *RUNX1* expression rate in skin biopsies of dcSSc patients (*N*=11) and healthy donors (*N*=5) from the Pendergrass *et al*., 2012 study (GSE32413). (C) *RUNX1* expression rate in skin biopsies of dcSSc patients (*N*=8) and healthy donors (*N*=4) from the Franks *et al.*, 2019 study (GSE125362). (D) *RUNX1* expression rate in skin biopsies of dcSSc patients (*N*=18) at baseline and a 12-month follow-up from the Gordon *et al.*, 2018 study (GSE97248). (E) *RUNX1* expression rate in skin biopsies of dcSSc patients (*N*=43), lcSSc patients (*N*=18), and healthy donors (*N*=36) from the Assassi *et al.*, 2015 study (GSE58095). (F) Correlation between *RUNX1* expression and mRSS skin score at baseline for both lcSSc (yellow) and dcSSc (red) patients. (G) *RUNX1* expression rate to the skin score at the site of biopsy.


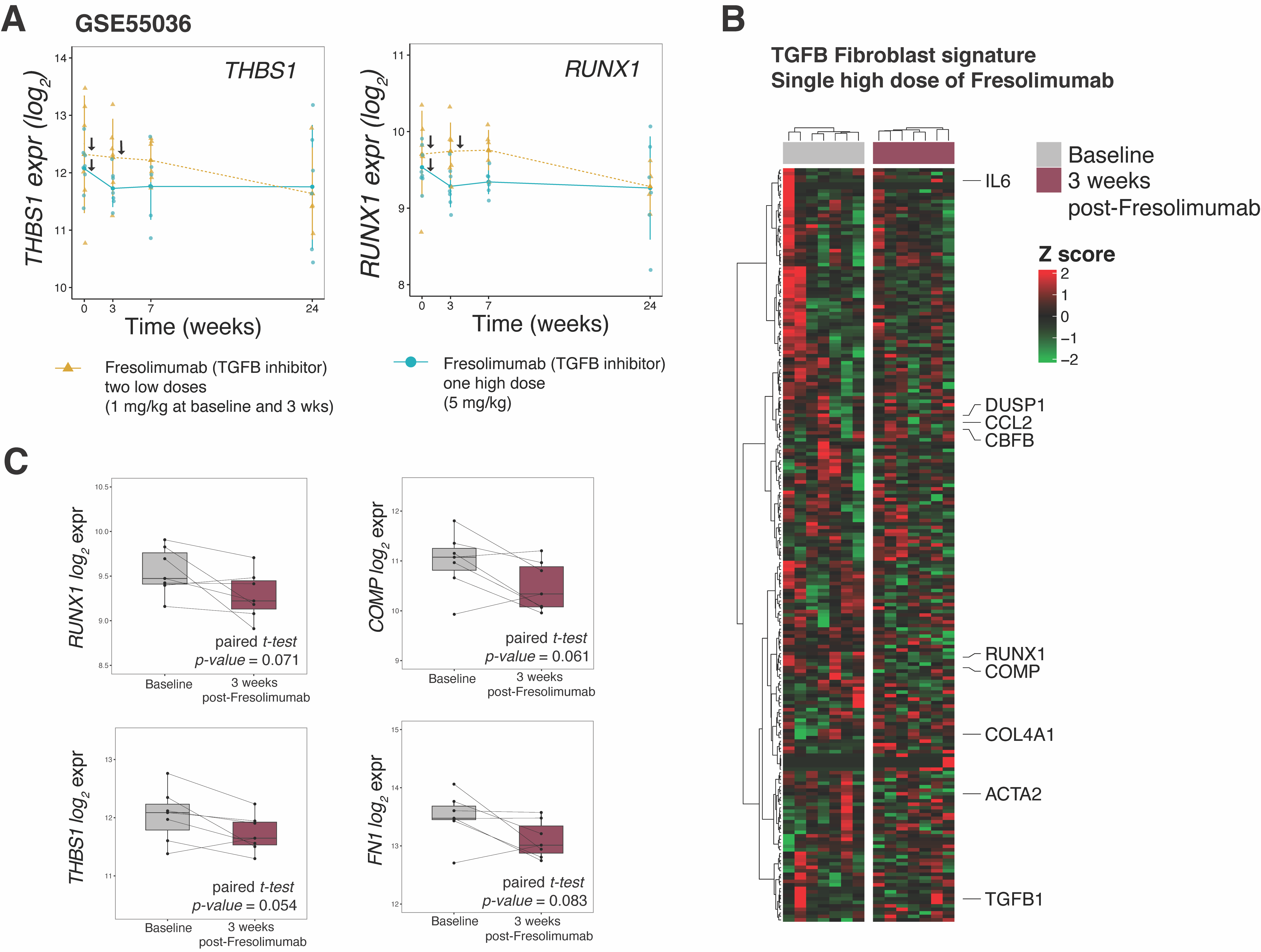


**Supplemental Figure 3.** **The** **TGF-β inhibitor fresolimumab reduces *RUNX1* expression in SSc skin.** (A) *THBS1* and *RUNX1* expression levels in dcSSc skin biopsies of patients who were given two low doses (1 mg/kg) of fresolimumab at weeks 1 and 3 in yellow (*N*=7); or a single high dose (5 mg/kg) of fresolimumab at week 1 in blue (*N*=7). The mid-forearm skin biopsies were collected at baseline and again at weeks 3, 7, and 24. (B) The heatmap of genes in the TGF-β fibroblast cell signature for patients who received a high dose of fresolimumab at baseline and again 3 weeks after treatment (*N*=7). (C) The expression of several genes including *RUNX1* and TGF-β pathway biomarkers such as *COMP*, *THBS1*, and *FN1*.


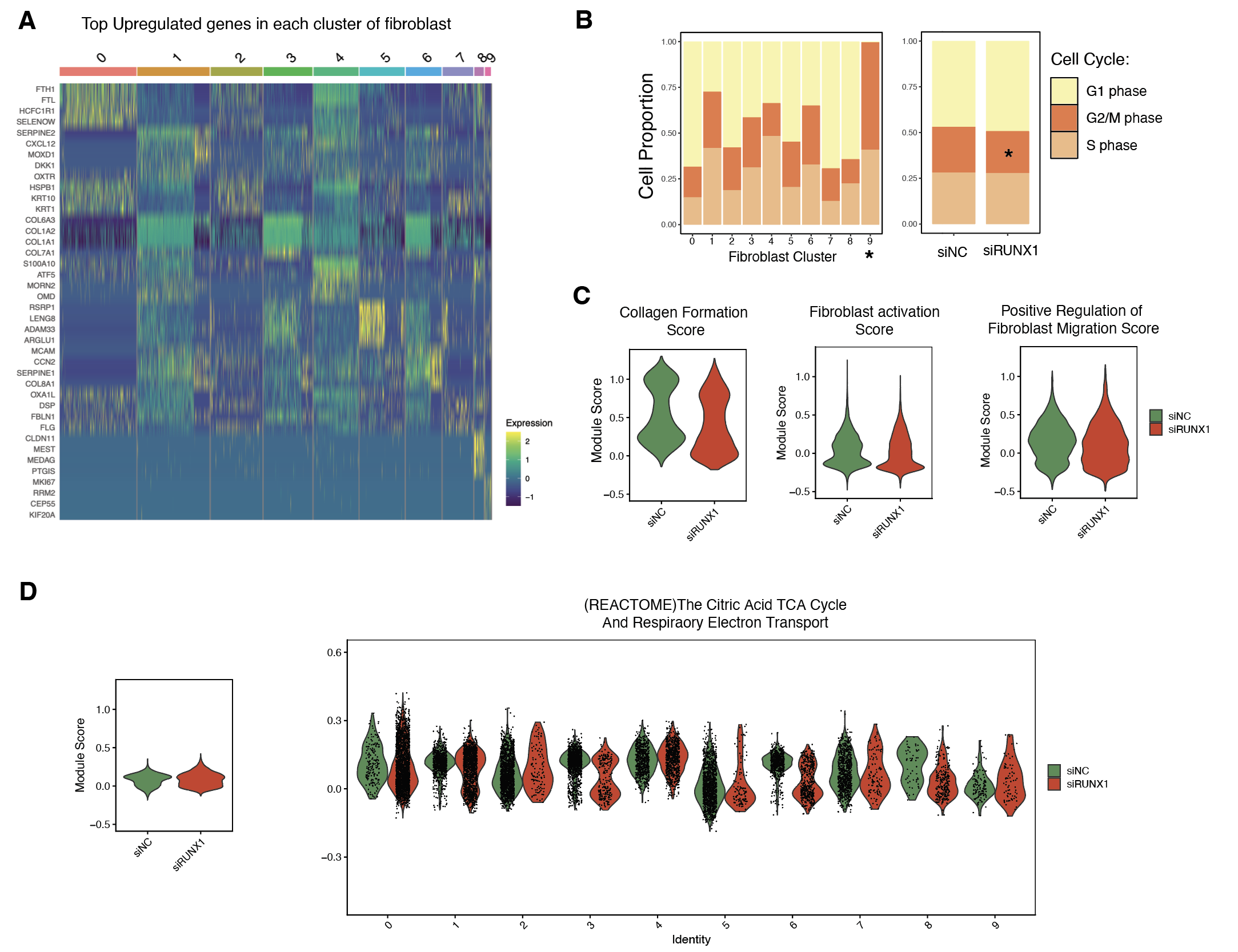


**Supplemental Figure 4.** **RUNX1 Knockdown in SSc fibroblast.** (A) Heatmap showing the top marker genes for each fibroblast cluster. (B) Proportion of cells in G1, G2/M, and S cell cycle phases in each cluster and treatment condition. P-value of Chi-squared test = 0.008 for all phases, and p-value = 0.005 for G2/M siRUNX1 vs siNC. (C) Module scores for collagen formation, fibroblast activation, and positive regulation of fibroblast migration gene sets. (D) Module scores for the Citric Acid TCA Cycle and Respiratory Electron Transport, that is lower for siRUNX1 samples particularly in cluster 0, 6, and 8.


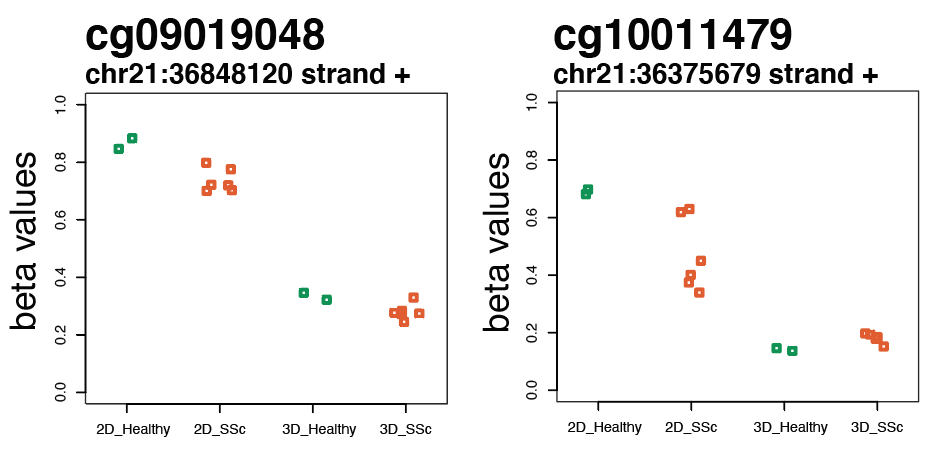


**Supplemental Figure 5.** The beta values of representative CpGs in *RUNX1* locus in 2D and 3D SSc and healthy conditions.


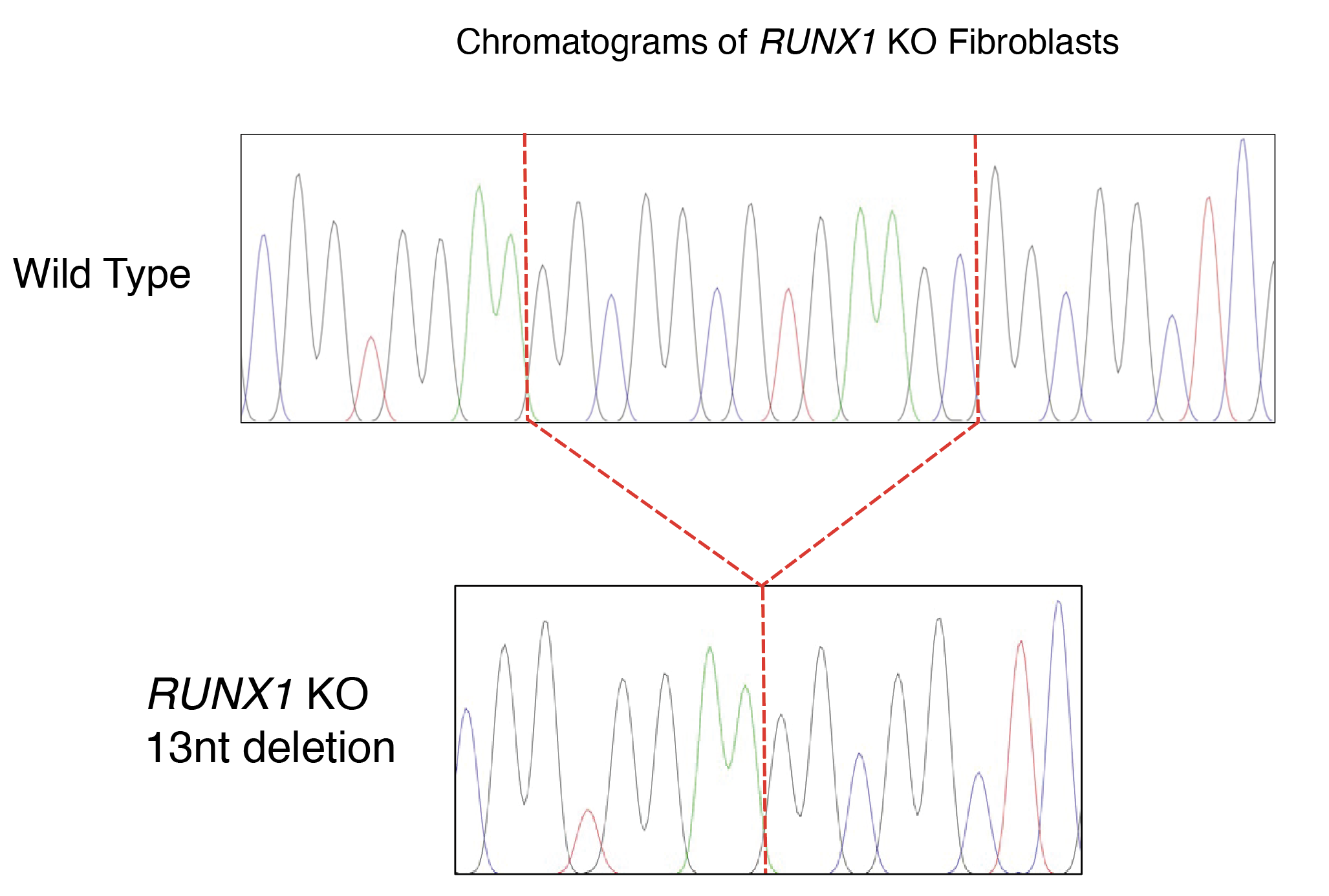


**Supplemental Figure 6.** Chromatograms of the homozygous RUNX1 KO fibroblast line and the wild type.

**Supplemental Figure 7.** Fold change mRNA expression of *FN1* and *COL1A1* in 10, 20, and 50 µM of Ro5-3335 treated SSc Fibroblasts.
